# Biased inter-columnar communication and short-term plasticity in mouse barrel cortex

**DOI:** 10.1101/2025.11.04.686617

**Published:** 2025-11-06

**Authors:** John M. Judge, Meyer B. Jackson

**Affiliations:** 1 Biophysics PhD Program, U. of Wisconsin-Madison, Wisconsin Institutes for Medical Research, 1111 Highland Ave, Madison, WI 53705; 2 Dept. of Neuroscience, U. of Wisconsin-Madison, Wisconsin Institutes for Medical Research, 1111 Highland Ave, Madison, WI 53705

## Abstract

The barrel cortex (BC) processes input from whiskers to probe and analyze objects in an environment. This sensory input exhibits a complex phase, direction, and frequency-dependent structure arising from whisking kinematics. Little is known about how BC microcircuits process this information. In particular, it remains unclear how the BC extracts relevant spatiotemporal features by integrating input from multiple whiskers. To investigate communication within and between cortical barrels, we targeted a hybrid voltage sensor (hVOS) to Scnn1a excitatory neurons in BC layer 4 (L4) of male and female mice (mean age 7.8 weeks), and imaged population responses to electrical stimulation. Coronal and sagittal slices presented the laminar structure with barrels aligned along stereotyped whisking directions. Voltage imaging tracked activity along an L4→L2/3→L4 relay during inter-barrel communication. AMPA receptor blockade demonstrated that this relay depends on excitatory synaptic transmission and revealed intra- and inter-barrel feedforward inhibition. Single-pulse responses were isotropic in amplitude, conduction velocity, and half-width, but latency was longer for communication with dorsal and caudal barrels. Furthermore, paired-pulse depression was weakest and recovery slowest for protraction-related directions, especially between caudally adjacent barrels, suggesting preferential enhancement of repetitive inputs in this direction. These results identify direction-dependent synaptic circuitry in the shaping of inter-barrel communication. Anisotropy in short-term plasticity aligns with whisker motion kinematics, suggesting that BC microcircuits are tuned to preserve temporal fidelity and selectively filter inputs according to whisking phase and direction.

## Introduction

The somatotopic map of mystacial whiskers in rodent primary somatosensory cortex serves as a model system for modular information processing by cortical circuits (Egger et al., 2012; Stüttgen & Schwarz, 2018; Woolsey, 1970). Each whisker maps primarily to one barrel within the barrel cortex (BC). These columnar *barrel* structures are visually distinct in cortical layer 4 (L4), which lacks the extensive inter-barrel connections that characterize layer 2/3 (L2/3) and layer 5 (L5) (Feldmeyer, 2012). Inter-barrel connections spread the activity elicited by a single whisker deflection across the entire barrel field (Petersen et al., 2003; Vilarchao et al., 2018). The velocity of this spread is neither constant nor isotropic; it is twice as rapid along whisker rows as along arcs (Petersen et al., 2003). Denser arborization in L2/3 along rows than arcs may support anisotropic processing (Adesnik & Scanziani, 2010; Feldmeyer, 2012; Staiger & Petersen, 2021). While whisker input follows the canonical thalamocortical path (Lübke et al., 2000; Viaene et al., 2011), less is known about how communication between barrels contributes to sensory processing. Previous work has examined *in-vivo* flow of sensory information (Armstrong-James et al., 1992; Zhang & Alloway, 2005) and spontaneous activity (Reyes-Puerta et al., 2016) between layers and barrels. However, corticothalamic feedback makes it difficult to isolate thalamic and cortical contributions to multi-whisker integration (Ma & Patel, 2021; Simons, 1985). *Barreloids* in ventroposterior medial thalamus (VPM) project to BC L4 (Agmon & Connors, 1991; Landisman & Connors, 2007) and receive feedback from L6 (Kwegyir-Afful et al., 2005; Lam & Sherman, 2009). The thalamocortical pathway projects to multi-barreloid receptive fields and provides feedforward inhibition onto BC (Fox et al., 2003; Simons, 1978; Sun et al., 2006). This corticothalamic feedback complicates the relationship between thalamic and cortical multi-whisker processing (Patel, 2019).

These structures play interdependent roles in frequency-specific transformations and temporal filtering. Thalamic firing rate encodes features of whisker kinematics, including whisking frequency (Aguilar & Castro-Alamancos, 2005; Petersen et al., 2008). The VPM firing rate increases sub-linearly (32-88 Hz) with whisker deflections at 2-11 Hz (Sosnik et al., 2001). This supports a dynamic range from 1-5 Hz exploratory whisking up to small-angle 15-25 Hz foveal whisking (Adibi, 2019). Intracortical synapses may implement temporal filtering (Fortune & Rose, 2001) to operate on thalamocortical spiking frequencies of 30-140 Hz. Furthermore, these preferences vary across the somatotopic map reflecting whisker specialization such as frequency-specific tasks of short, thin rostral whiskers versus longer, thicker caudal whiskers (Hobbs et al., 2016; Woolsey, 1970). While whisking occurs predominantly along the rostral-caudal axis, variation between foveal and exploratory whisking contributes to directional preferences (Knutsen et al., 2005; Petersen et al., 2020). In the barrel maps of primary and secondary somatosensory cortex, caudo-dorsal whiskers induce stronger responses than rostro-ventral whiskers (Hubatz et al., 2020). Additionally, BC responds preferentially to multi-whisker deflection directed caudo-ventrally (Vilarchao et al., 2018), suggesting asymmetry in inter-barrel communication. Although *in-vivo* studies describe preferred whisking phases for information gathering (Bermejo et al., 2002; Curtis & Kleinfeld, 2009; de Kock & Sakmann, 2009; Kleinfeld et al., 2016; Leiser & Moxon, 2007), the underlying inter-barrel microcircuits supporting these preferences remain undetermined.

Experiments *in vitro* in brain slices provide an opportunity to study intra-cortical communication in isolation, and have shown that electrical stimulation evokes activity that propagates from L4 to supragranular layers before spreading to other barrels (Hill & Greenfield, 2014; Laaris & Keller, 2002; Petersen & Sakmann, 2001; Sato et al., 2008). Here we targeted a hybrid voltage sensor (hVOS) to L4 excitatory neurons (spiny stellate and pyramidal) in mouse BC slices to image voltage in these neurons that receive the primary thalamic input. Excitatory synapse blockade isolated barrels and revealed a dependence on synaptic transmission in L2/3 and L4 for communication between barrels. We investigated response characteristics, velocities, and synaptic depression in BC microcircuits. Strategic choices of slice plane preserved specific inter-barrel connections, providing insight into isotropic and anisotropic aspects of inter-barrel communication.

## Materials and Methods

### Animals.

Ai35-hVOS1.5 (C57BL/6-Gt(ROSA) 26Sortm1 (CAG-hVOS1.5)Mbja/J; JAX #031102) Cre reporter mice were bred with Scnn1a-tg3-Cre driver mice (B6;C3-Tg(Scnn1a-cre)3Aibs/J; JAX # 009613) to create animals with hVOS probe expression (Bayguinov et al., 2017; Chanda et al., 2005) specifically in L4 excitatory neurons (Madisen et al., 2009; Scala et al., 2019). All animal procedures were approved by the Animal Care and Use Committee of the University of Wisconsin-Madison School of Medicine and Public Health (IACUC protocol: M005952).

### Slice preparation.

Male and female mice were deeply anesthetized with isoflurane and sacrificed via cervical dislocation. Brains were dissected and placed into ice-cold cutting solution (in mM: 10 glucose, 125 NaCl, 4 KCl, 1.25 NaH_2_PO_4_, 26 NaHCO_3_, 6 MgSO_4_, 1 CaCl_2_) bubbled with 95% O_2_/5% CO_2_. Coronal (~6 per hemisphere per animal) and sagittal (~4 per hemisphere per animal) slices 200 μm in thickness were prepared using a Leica VT1200S vibratome and placed into artificial cerebrospinal fluid (ACSF, same as cutting solution except 1.3 mM MgSO_4_, 2.5 mM CaCl_2_) and bubbled with 95% O_2_/5% CO_2_ for at least 1 h. The ACSF used for slice storage and recording contained 4 μM DPA (Scheuer et al., 2023).

### Voltage imaging and electrical stimulation.

Slices were continuously perfused with ACSF at room temperature and viewed using an Olympus BX51 microscope and Olympus XLUMPlanFl 20 × objective (N.A. 1.0). Layer and barrel boundaries were visually identified in both fluorescence and gradient contrast images. Stimulus pulses (50-250 μA, 200 μs) were generated by triggering a stimulus isolator (World Precision Instruments, Sarasota, FL) and applied via a fire-polished, ACSF-filled KG-33 glass microelectrodes (King Precision Glass, Claremont, CA) with tip diameters ~6–8 μm. Displayed traces of fluorescence versus time were averages of five trials, at 15 s intervals. Slices were illuminated with a light emitting diode (Prizmatix, Holon, Israel) with peak emission at 435 nm. Gradient contrast images were acquired with a high-resolution Kiralux CMOS camera (Thorlabs, Newton, NJ) for better visualization. For voltage imaging we used a da Vinci 2K CMOS camera with a frame acquisition speed 2 kHz binned to 80 × 80 spatial resolution (RedShirt Imaging, Decatur, GA). A movable mirror within a dual-port adapter was used to switch between the high-resolution Kiralux CMOS camera and the high-speed da Vinci 2K camera. Image acquisition is controlled by the TurboSM software (RedShirt Imaging / SciMeasure) and a custom application to interface TurboSM with an in-house C++ application for data processing (PhotoZ; Chang et al., 2006). To extend stimulation protocol flexibility we coupled TurboSM with a programmable pulse train generator (Prizmatix). We developed a separate in-house Python application (OrchestraZ) to coordinate these three software applications into a single workflow.

### Analysis, Statistics.

We analyzed data from 48 barrels in 21 slices from 11 male mice and 39 barrels in 16 slices from 8 female mice aged 4-13 weeks (mean ± SD age 7.8 ± 1.7 weeks, 2-3 barrels per slice, 1-4 slices per animal). A comparison of male versus female mice showed no significant differences in latency, half-width, velocity (Welch’s t-test, T < 0.001, p = 1.0 for all), or paired-pulse ratio (PPR) (multivariate linear regression, F=0.55, p =0.46). Mice aged 30 to 60 days showed no significant correlation between these metrics and age: response latency, half-width (linear regression, L2/3: latency: p = 0.23, half-width: p = 0.48; L4: latency: p = 0.16, half-width: p = 0.57), velocity (linear regression, p = 0.66) or PPR (multivariate linear regression, F = 0.006, p = 1.0).

PhotoZ implements baseline subtraction, temporal and spatial filtering, and linearly-interpolated measurements of latency, half-width, and peak ΔF/F measurements. The latencies are times from the stimulation pulse to half-amplitude. Time windows for analysis were from 3 ms before stimulation to 50 ms post-stimulation, with independent samples obtained as barrel-averaged measurements. Domains with lengths of at least 50 μm showing constant velocity were identified for linear regression and velocity measurement. Velocity was calculated with the SciPy stats Python library by performing linear regression on latency versus distance along L4, using 7 or more regions of interest (ROIs) in a single barrel. Pixels within 50 μm of the stimulation site were excluded, affecting only stimulated (home) barrel measurements. Septa between barrels were also excluded due to their low hVOS probe expression and dim fluorescence.

We tested multiple groups for significance with one-way or two-way ANOVA implemented by the SciPy stats Python library. For pairwise comparisons after ANOVA tests, Shapiro-Wilk tests confirmed normality before we used Tukey’s honestly significance difference (HSD) test, implemented by the Statsmodels Python library. We confirmed that inter-class variance was not significantly greater than intra-class variance with a mixed-effect linear regression model implemented in R, obviating the need to control for pseudoreplication effects between slices of the same animal or litter (Lazic, 2010). In cases where only two groups were compared, we used the one-sided Welch’s t-test from the Scipy.stats Python library. We also used the one-sample one-sided t-test from Scipy.stats to compare mean against the appropriate null hypothesis value.

To test the dependence of measurements on direction we hypothesized that the differences between dorsal minus ventral and rostral minus caudal will share the same sign because ventral and caudal neighbor activations are associated with whisker protraction, whereas dorsal and rostral neighbor activations are associated with retraction, taking the angular offset between BC and whisker field into account. Because each group is comprised of independently measured barrels, we used a contrast coding system on linear regression (using the Patsy Python library for sum contrast coding with Statsmodel’s ordinary least squares function). Using this sum contrast coding (comparing to the group mean as reference), we tested whether the differences of each pair of group means (represented by μi) would sum to significantly greater than zero on average (one-sided t-test); that is, our contrast model alternative hypotheses were:

$$(\mu_{dorsal}-\mu_{ventral})+(\mu_{rostral}-\mu_{caudal})>0$$

This tested for protraction < retraction; signs were flipped to test for protraction > retraction. The contrast model allows for a single, specific, higher-powered t-test without the family-wise error rate corrections for the 6 corresponding pairwise tests in Tukey’s HSD test.

We designed our sample sizes to detect intracortical processing differences on the order of at least 15%. To achieve a standard power level (0.8) and false positive rate of 5%, allowing for an uncertainty of 8%, we calculated that a minimum sample size of 8 is needed: $n_{2}=n_{1}=\frac{\sigma_{1}^{2}+\sigma_{2}^{2}∕K}{\Delta^{2}}{\left(z_{1-\alpha ∕2}+z_{1-\beta ∕2}\right)}^{2}=4$. For post-hoc power analysis, we measured variances (ΔF/F ratios: 14%, half-width: 25%, velocity: 19-26%, latency: 10-19%, PPR parameters: 20-50%) and calculated effect sizes and sample sizes based on the contrast model (reported in Results), which are similar to those of the sagittal-coronal comparison. Tests of half-width (0.15), ΔF/F ratios (0.17), and latency (0.54) were underpowered. The power levels of velocity (1.0) and PPR at 20 ms interpulse interval (IPI) (0.73) indicated a sufficient sample size to achieve standard statistical power.

## Results

We first present a characterization of barrel organization in coronal and sagittal slices as revealed by Scnn1a labeling, and evaluate the alignment between barrels in our slices and whiskers. We then use voltage imaging to follow the spread of excitation within and between barrels, evaluate the contributions of excitatory synaptic transmission, and present results in support of the view that signaling between L4 of neighboring barrels employs an L4→L2/3→L4 relay. We evaluate response parameters of inter-barrel communication and conduction velocity in different directions. Finally, we integrate voltage imaging results with barrel alignment in order to assess orientation biases in signaling and short-term plasticity between barrels along behaviorally relevant axes.

### Barrel visualization and alignment

**Fig. 1:**
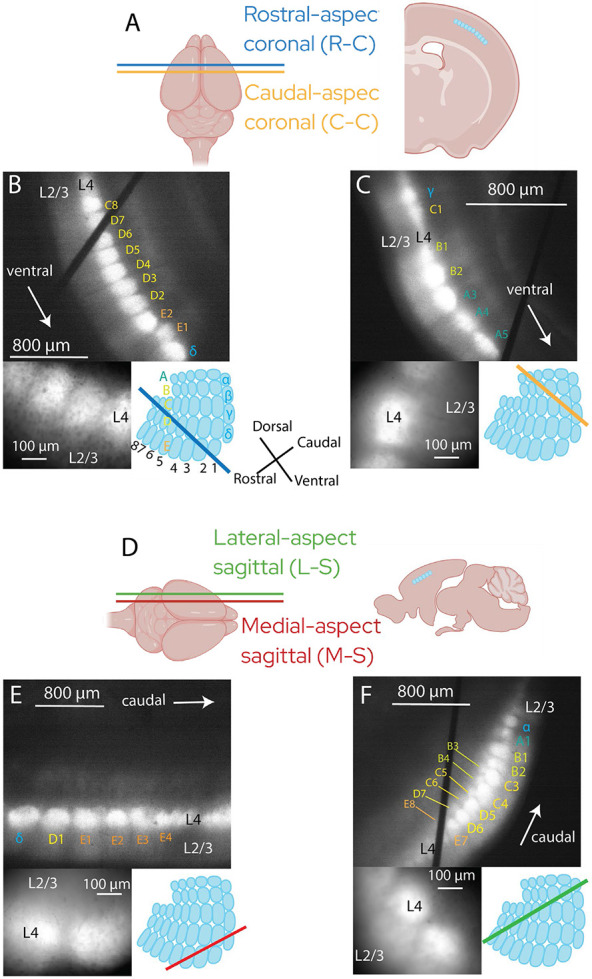
Anatomical orientation and slicing strategy. **A.** Left: dorsal view of mouse brain in the coronal plane: rostral-aspect coronal (R-C, blue) and caudal-aspect coronal (C-C, orange). Right: Coronal slice with barrels highlighted as blue (created with Biorender). **B-C**. Top: low-magnification fluorescence and gradient contrast images superimposed from R-C (B) and C-C (C) slices, with annotations for barrel, barrel relationship, and orientation. Lower left: higher magnification of the corresponding slice. Lower right: diagram of tangential section of barrel cortex; blue and orange lines indicate approximate location of respective slicing planes for R-C and C-C in the barrel field. **D.** Left: Dorsal view of mouse brain showing slicing planes: lateral-aspect sagittal (L-S, green) and medial-aspect sagittal (M-S, red). Right: illustration of barrels in a sagittal slice (created with Biorender). **E.** M-S slices; **F**. L-S slices, paralleling panels and annotations in B and C. Barrel row and arc assignments are given to within one row or arc; this uncertainty was acceptable because determining only aspect and plane classification are needed.

The columnar organization of the BC is clearly visible in the pattern of fluorescence in somatosensory cortex slices from Scnn1a::hVOS mice, as illustrated with low magnification fluorescence images (Fig. 1). Labeling is concentrated in L4 excitatory neurons targeted by Scnn1a, and is stronger in barrel hollows than septa. We used the Brain Globe 3D atlas of mouse barrel cortex (Claudi et al., 2020) to align barrels in our slices with rows and arcs of whiskers, and used this alignment to guide our voltage image experiments in the evaluation of directionality in inter-barrel communication. Our coronal slices preserved dorsal-ventral connections (Fig. 1A-C) and our sagittal slices preserved rostral-caudal connections (Fig. 1D-F). However, the planes of slicing cannot strictly preserve rows and arcs due to the curvature of the barrel field grid and a rotation from the whisker grid by what we estimate at up to ~35 degrees from the 3D atlas. This is consistent with typical medial-lateral angles used to cut tangential BC slices (Sumser et al., 2017). Adjacent barrels are usually in the same row or arc, but occasionally are in different rows or arcs. To address these uncertainties, we divided our slices into groups based on aspect and orientation to account for barrel relationships.

Each panel of Figs. 1B, 1C, 1E, and 1F shows a low magnification image with barrel relationships annotated (top), accompanied by a higher magnification image (bottom left) and a tangential view of BC with slice plane drawn as a thick colored line (bottom right). Thus, the connections that are preserved depend not only on the plane of section (coronal versus sagittal) but also on slice location of origin (e.g. coronal sections taken from the rostral versus caudal aspects, shown in Fig. 1A). On this basis we divided slices into four groups: rostral-aspect coronal (R-C slice, Fig. 1A, 1B), caudal-aspect coronal (C-C slice, Fig. 1A, 1C), lateral-aspect sagittal (L-S slice, Fig. 1D, 1E), and medial-aspect sagittal (M-S slice, Fig. 1D, 1F). R-C slices tend to present a cross-section of rows C, D, or E (labeled in Fig. 1B, bottom right), which curve into the rostro-dorsal direction in the rostral aspect. (Fig. 1A, right). These rows are visually distinguished by their narrow septa and higher barrel count (7-10 per slice). In contrast, C-C slices tend to present fewer, larger barrels and septa (Fig. 1C). Sagittal slices originated from both lateral and medial aspects (Fig. 1E: M-S, Fig. 1F: L-S), with lateral slices containing barrels corresponding to ventral whiskers and medial slices containing barrels corresponding to dorsal whiskers (Petersen et al., 2020). In L-S slices, however, the slice plane becomes nearly tangential, such that cross sections of multiple barrels stack along the cortical column axis; two rows or arcs of barrels become visible within L4 (Fig. 1F). This contrasts with the perpendicular layer-barrel alignment preserved in the other three types of slices. Because our goal is distinguishing inter-barrel connections, we excluded L-S slices from the present study.

**Fig. 2:**
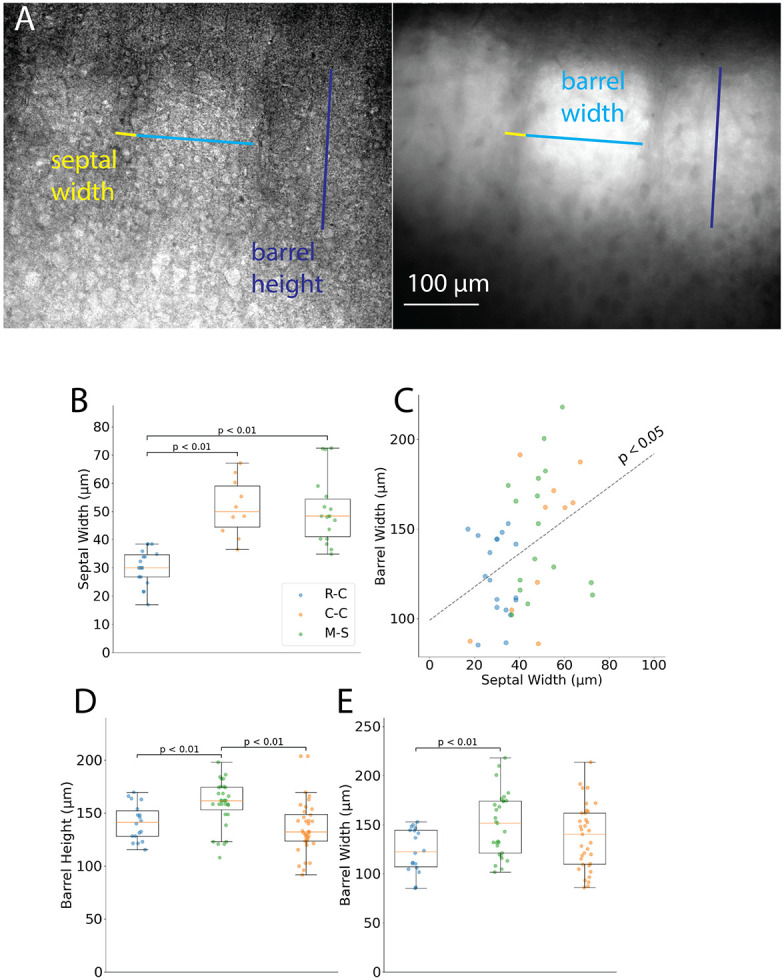
Barrel dimensions across slice types. **A.** Bright field (left) and resting fluorescence (right) images featuring barrel width (light blue) and height (dark blue), and septum width (yellow). The L2/3-L4 boundary is determined based on the presence of septal columns and higher fluorescence in L4 than in L2/3. **B**. Comparison of septal width among R-C, C-C, and M-S slices (note that L-S slices were not included, see text). **C**. Plot of barrel width versus septum width with line from linear regression (p < 0.05). **D-E.** Barrel height and width differ across slice types (M-S barrel height is greater than that of R-C and C-C; R-C barrels were narrower than M-S barrels). Each point indicates one barrel or septum; lines show mean ± SEM. Significance assessed by Welch’s t-test.

The clear boundaries highlighted by Scnn1a labeling enabled us to estimate the basic barrel dimensions. The septal width, barrel width, and barrel height, annotated on bright-field (left) and fluorescence (right) images in Fig. 2A, were estimated from the cytoarchitectural boundaries. We distinguished L2/3 from L4 based on a steep fluorescence gradient and the loss of distinction between septa and barrel hollows at the boundary. Fig. 2B compares septal widths between the R-C, C-C, and M-S slices. The mean septal width in the rostral row of R-C slices, 30.3 ± 1.5 μm (n = 18 septa, 15 slices), was significantly smaller than in C-C slices (51.5 ± 3.2 μm, n = 10 septa, 10 slices; Welch’s t-test: T = 5.99, p = 5x10^−5^) or in M-S slices (50.4 ± 2.8 μm, n = 18 septa, 13 slices, T = 6.38, p = 10^−6^). R-C and M-S septal widths did not differ significantly (T = 0.24, p = 0.8). These variations were exploited to track the transition from R-C to C-C in the sequence in which slices were cut. Barrel width and height did not correlate significantly (linear regression, R = 0.19, p = 0.09), which is expected given the heterogeneity of barrel dimensions and the fact that the thickness of L4 limits barrel height to 208 ± 5 μm or less (Defelipe et al., 2002). However, barrel and septal width showed a significant positive correlation (Fig. 2C, R = 0.38, p = 0.01). Additionally, sagittal slices were cut at a smaller angle (less than a right angle) to the cortex layers, particularly towards the medial aspect but also in the lateral aspect. As a result, L4 is thicker in some sagittal slices and barrel height is significantly greater in M-S slices (Fig. 2D, 159 ± 4 μm, n = 31 barrels) than in R-C slices (141 ± 4 μm, n = 18, T = 3.1, p = 0.003) or in C-C (137 ± 4 μm, n = 36, T = 3.9, p = 0.0003). Barrel widths were significantly smaller in R-C slices (Fig. 2E, 124 ± 5 μm, n = 18) than in M-S slices (150 ± 6 μm, n = 29, T = 3.33, p = 0.002), but not significantly different from C-C slices (138 ± 6 μm, n = 35, T = 1.55, p = 0.13). These differences in barrel width match the trend in barrel size seen in the tangential view of BC, in which barrels become smaller in the rostro-medial aspect and have smaller cross-sections in the coronal plane (Fig. 1B, lower right; (Petersen et al., 2020). Our choice of 200 μm slice thickness limits background from barrels outside of the microscope focal plane, given the sum of our measurements of barrel width plus septal width (154-200 μm).

We sampled the Brain Globe 3D atlas (Claudi et al., 2020) in 12 locations relevant to each group of slices to estimate the relative likelihood of adjacent barrels being in the same row or same arc. The relative likelihood of neighboring barrels being in the same row or arc was 0.90 in R-C slices, 0.50 in C-C slices, and 0.78 in M-S slices (excluding adjacent barrels of both different rows and different arcs). We originally intended to associate R-C slices and C-C slices with dorsal-ventral arc whisking and M-S slices with rostral-caudal row whisking. However, this atlas sample suggests associating R-C slices with rostral-aspect whisking, C-C slices with caudal-aspect whisking, and M-S slices with caudal-aspect whisking is more accurate. In addition, our sections align well to specific stereotyped whisking patterns. R-C and C-C slices track exploratory whisking, with protraction angles to the rostral-dorsal direction. The M-S plane tracks foveal whisking, with protraction angles toward the rostral-ventral direction (Knutsen et al., 2005; Petersen et al., 2020). This unexpected correspondence is a consequence of BC curvature and an angular offset between barrel and whisking fields. For example, the dorsal direction in the barrel field functionally corresponds to the rostral-dorsal direction in the whisker field. However, this offset offers a distinct advantage: the two slicing planes in this study track two perpendicular axes relevant to whisking (foveal and exploratory). Our alignment allows us to relate these whisker field axes to our imaging results on inter-barrel communication.

### Stimulation induced depolarization of multiple barrels

**Fig. 3:**
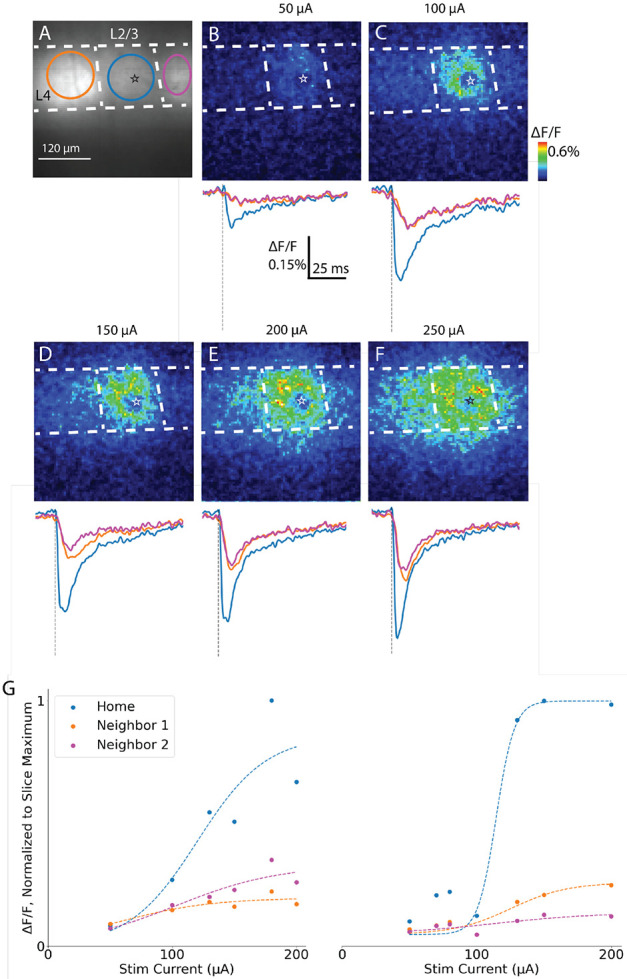
Stimulation-induced voltage changes in barrel cortex slices. **A.** Resting fluorescence image of coronal slice with L4 stimulation site marked with a star. ROIs within home and neighbor barrels are delineated with colored boundaries. **B-F.** Maps of peak ΔF/F in response to increasing stimulation currents (color scale denotes response amplitude). Traces below each panel show fluorescence versus time for each ROI (colors of traces correspond with ROI outlines). Spatial scale in A applies to all images. **G**. Plots of peak ΔF/F versus stimulation current from two different experiments for home and neighbor barrels. Plots were fitted with sigmoid curves (see text, dotted lines) for home and neighbor barrels.

In both coronal and sagittal slices, electrical stimulation in L4 elicited fluorescence changes in both the *home* barrel (the barrel to which stimulation was applied), and the two *neighboring* barrels on either side of the home barrel (Fig. 3). A coronal slice shown in Fig. 3A was stimulated at the site indicated by a star with currents ranging from 50-250 μA in a random sequence. Maps with peak response amplitude (ΔF/F) encoded as color illustrate the spatial spread of depolarization for a sequence of increasing stimulation currents (Fig. 3B-F). To view the time course of responses at specific locations we selected ROIs outlined in Fig. 3A encompassing individual barrels within L4, and averaged the fluorescence within these ROIs to produce traces of fluorescence versus time (traces below each map). hVOS imaging registers a positive change in voltage as a decrease in fluorescence resulting from the movement of negatively charged DPA toward the fluorescent protein at the inner membrane face to quench emission (Chanda et al., 2005). Thus, these downward-going signals report depolarization of probe-expressing neurons. The traces show that responses in neighboring barrels have smaller amplitudes and slower time courses compared to the home barrel. The response amplitudes in the directly stimulated home barrel (blue traces) and neighbor barrels (orange and pink traces) increase as stimulation current increased, due to both increases in peak ΔF/F of individual pixels as well as spatial spread within the ROIs. In both home and neighbor barrels, peak ΔF/F tended to saturate with high stimulation current.

We plotted peak ΔF/F versus current and fitted the curves to a sigmoidal function of the form $\frac{\Delta F}{F}=a\;{\left(1+e^{-b(I-c)}\right)}^{-1}$ with stimulation current I. This expression yielded a good fit for 7 of 8 home barrels (home R^2^ = 0.93 ± 0.03) and 12 of 14 neighbor barrels (R^2^ = 0.88 ± 0.03), as illustrated with two examples in Fig. 3G (fits in dotted lines). The fitting parameter c represents the stimulation current required to elicit a half-maximal response, and was 94 ± 11 μA for home barrels and 126 ± 26 μA for neighboring barrels (difference not significant, Welch’s t-test, T = 1.1, p = 0.3). We used the sigmoidal fits to determine the 70% saturation currents, which averaged 129 μA and 178 μA, respectively. To investigate inter-barrel spread in more detail, we stimulated with roughly 70% of saturation current determined for each slice (ranging from 80-200 μA, mean 155 ± 9 μA) in order to achieve nearly full spatial spread while avoiding damage.

### Barrel responses to stimulation of different layers

**Fig. 4:**
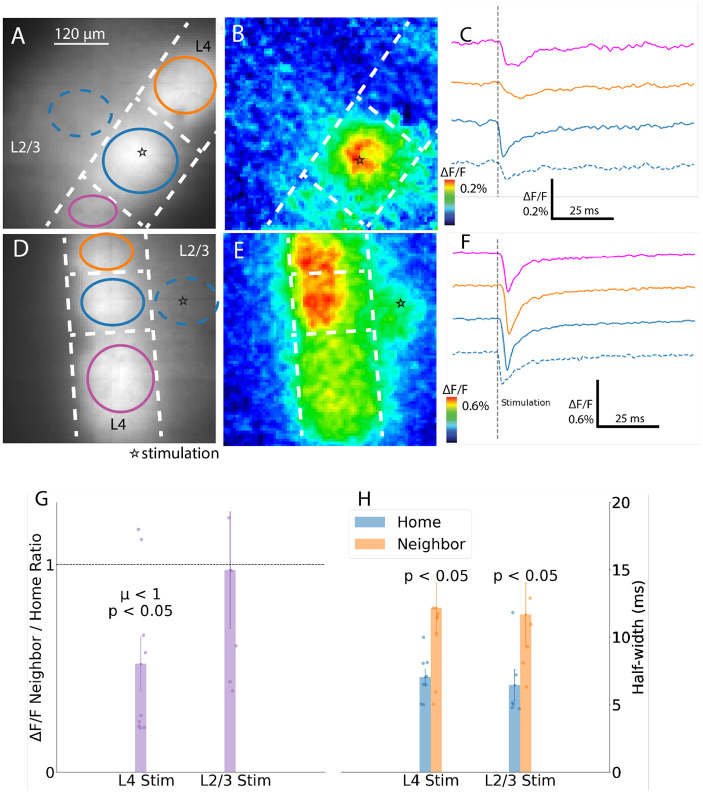
Response patterns for stimulation in L4 and L2/3. **A-C.** L4 stimulation evokes strong responses in home barrels and weaker responses in neighbors. **A**. Image of coronal slice with star marking the stimulation site. ROIs drawn within home and neighbor barrels. **B**. Map of peak ΔF/F (color scale denotes response amplitude; each map is scaled to its maximum). **C**. Traces of ROI-averaged fluorescence intensity with corresponding color and line style as in A. **D-F.** L2/3 stimulation elicits broader, nearly equal responses across barrels. D-F parallel A-C. **G.** Neighbor/home ratios of peak ΔF/F are significantly lower for L4 than L2/3 stimulation (n = 9 and 6 barrels). **H**. Neighbor barrel half-widths were significantly greater than home barrel for both stimulation sites. Bars: mean ± SEM with points for individual slices.

Responses varied depending on the site of stimulation. Response maps show that stimulation in L4 produced less spread than stimulation of L2/3 (compare Figs. 4A and 4B with Figs. 4D and 4E) and traces show responses in neighboring barrels had smaller amplitudes for L4 stimulation (Fig. 4C) versus L2/3 stimulation (Fig. 4F). Neighboring and home barrel responses had similar amplitudes with L2/3 stimulation, but neighboring barrels had smaller response amplitudes with L4 stimulation. The ratio of neighbor peak ΔF/F to home peak ΔF/F was significantly less than one with L4 stimulation, 0.52 ± 0.13 (Fig. 4G, 1-sample t-test on neighbor/home ΔF/F ratio:, T = 3.7, p = 0.003, n = 9 slices) but indistinguishable from one with L2/3 stimulation, 0.97 ± 0.28 (T = 0.09, p = 0.5, n = 6). However, the ratios for L2/3 and L4 stimulation were not significantly different from each other (one-sided, T = 1.5, p = 0.093). With both L4 and L2/3 stimulation, the response in the neighboring barrel had a significantly greater half-width by almost a factor of two (Fig. 4H, Welch’s t-test, L4 stimulation, home: 8.8 ± 0.7 ms, neighbor: 15.2 ± 2.3 ms, T = 2.7, p = 0.01; L2/3 stimulation, home: 8.1 ± 1.4 ms, neighbor: 14.6 ± 2.9 ms, T = 2.0, p = 0.04). We also saw small fluorescence changes in L2/3 (dotted blue for the home barrel in Fig. 4E). Because the probe is expressed in neurons with somata located in L4 (Madisen et al., 2009), these responses in L2/3 arise from the depolarization of the axons and dendrites of our targeted Scnn1a neuronal subpopulation.

### AMPA-receptor blockade isolates postsynaptic responses

**Fig. 5:**
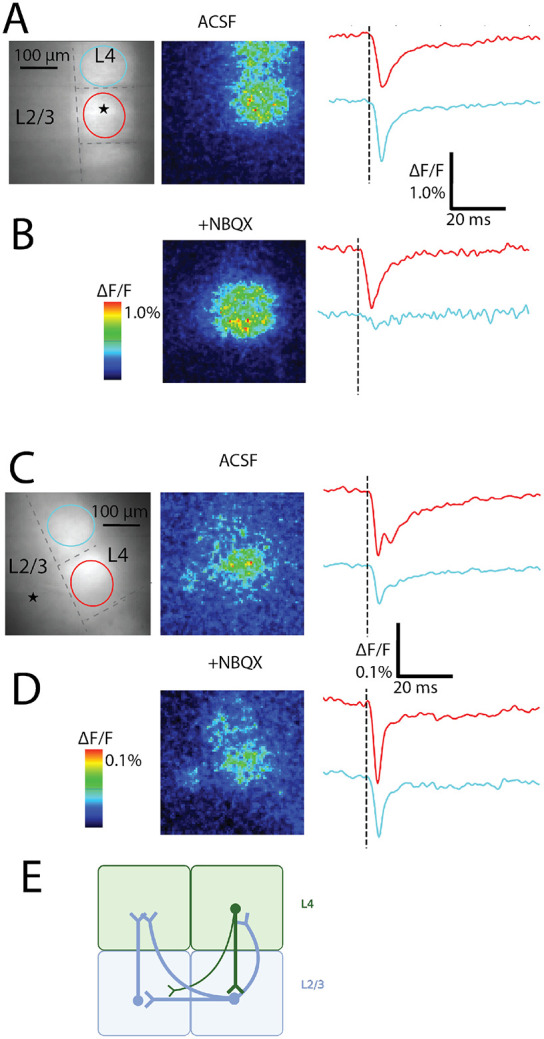
AMPA receptor blockade removes synaptic response components. **A.** L4 stimulation elicits responses in home and neighbor barrels. Left: resting fluorescence image of coronal slice with L4 stimulation site and ROIs annotated. Center: Map of peak ΔF/F. Color scale denotes response amplitude. Right: Traces of fluorescence versus time for each ROI of the corresponding color in the left panel. Vertical black dashed line: stimulation time. **B.** NBQX eliminated neighbor barrel responses, and slightly increased home barrel responses. Color scale applies to maps in A and B. Center/right panels parallel those in A. **C-D.** With L2/3 stimulation, NBQX had little effect on amplitude but reduced half-width. C-D parallel A-B. **E.** Circuit diagram: L4→L2/3→L4 mediates inter-barrel spread from L4 stimulation via monosynaptic or disynaptic pathways. Processes of Scnn1A neurons in L2/3 are activated antidromically by L2/3 stimulation. Thickness of axons in the diagram reflects the density of these connections in reconstruction studies (Staiger & Petersen, 2021) (Created with Biorender).

The spread of excitation illustrated in Figs. 3 and 4 could reflect direct depolarization, or activation, or orthodromic responses. Because orthodromic responses depend on excitatory synapses, we can assess their contribution by blocking AMPA receptors with the antagonist NBQX (10 μM). Fig. 5A presents a response map and fluorescence traces to illustrate the depolarizations in both the home and neighboring barrels elicited by L4 stimulation. NBQX nearly eliminated the depolarization of the neighboring barrel (Fig. 5B, left), reducing the peak amplitude by more than 5-fold (Fig. 5B, right). The strong blockade of neighboring barrel responses to stimulation in L4 demonstrated that these depolarizations depend on AMPA receptor-mediated excitatory synapses. By contrast, NBQX produced a modest increase in response amplitude within the home barrel (Fig. 5B). Thus, home barrel responses did not depend on excitatory receptors, and the increased amplitude indicated local inhibitory interneurons activated by excitatory synapses limited the responses seen in ACSF alone. NBQX had almost no inhibitory action on either home or neighbor barrel peak response amplitudes when L2/3 was stimulated, indicating L2/3 stimulation elicits predominantly antidromic action potentials (Fig. 5C-D).

We interpret these results with a minimal circuit (Fig. 5E) in which L4 stimulation first directly activates excitatory neurons in L4 of the stimulated barrel. These neurons then excite non-Scnn1a excitatory neurons with cell bodies in L2/3, which in turn excite Scnn1a L4 excitatory neurons in the neighboring barrel. This circuit illustrates how L4 activation of L4 in neighboring barrels uses L2/3 as a relay. Responses of neurons residing in L2/3 to L4 stimulation depend on excitatory synaptic transmission at L4→L2/3 synapses or antidromic activation of the axons of L2/3 neurons extending into L4. L2/3 neurons thus activated then excite L2/3 and L4 neurons in the neighboring barrel. Axons from L4 neurons are reported to pass through L2/3, cross into the adjacent barrel, with some then curving back up to L4. However, inter-barrel axons originating in L2/3 vastly outnumber these direct L4→L4 inter-barrel axons (Staiger & Petersen, 2021), consistent with the strong blockade by NBQX of L4 evoked neighboring L4 responses observed here. On the other hand, L2/3 stimulation directly activates the extensive inter-barrel arborization in this layer, and these axons conduct action potentials antidromically to L4 neurons. L2/3 stimulation evokes orthodromic synaptic responses as well; the briefer duration and loss of the second phase after addition of NBQX (Fig. 5D) indicate that AMPA receptors in L4 are activated by L2/3 stimulation, with this wave following the antidromic responses.

**Fig. 6:**
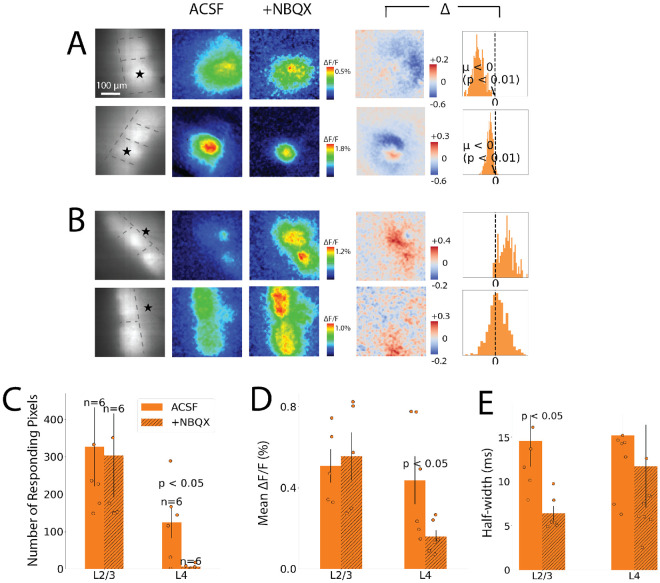
Spatial patterns of NBQX action. **A-B.** Each row corresponds to one slice before and after adding NBQX, with L4 stimulation (A) and L2/3 stimulation (B). Left-most column: resting fluorescence images with stimulation site (star) and layer/barrel boundaries (dashed lines). ACSF column: Maps of peak ΔF/F in ACSF. +NBQX column: Maps of peak ΔF/F after NBQX addition. Color scale matches maps in ACSF column (difference scale for each row). Δ column, left: Difference maps (Δ = NBQX – ACSF) show suppression in neighbor barrels for L4 but not L2/3 stimulation. White pixels represent zero change; red pixels represent responses increased by NBQX, and blue pixels represent responses reduced by NBQX. Δ column, right: : histograms of pixelwise differences for neighboring barrel only. Means were significantly less than zero for L4 stimulation (A), but not for L2/3 stimulation (B) (one-sample t-test). **C.** Responding pixels were defined as pixels in the neighboring barrel with peak ΔF/F above a slice-specific threshold. Response area (based on number of pixels) was decreased by NBQX for L4 but not for L2/3 stimulation. **D**. Mean peak ΔF/F was reduced by NBQX in neighbor barrels for L4 but not for L2/3 stimulation. **E.** Half-width before and after NBQX for neighbor barrels. Bars show mean ± SEM, with points for individual slices.

The traces in Fig. 5 were taken from ROIs that encompass nearly all of L4 between the hollows of the selected barrels. However, the heatmaps indicate that within these ROIs the action of NBQX varies spatially. To examine this variation, we constructed difference maps before and after adding NBQX, with two examples for stimulation in L4 (Fig. 6A) and two examples for stimulation in L2/3 (Fig. 6B). Difference maps, indicated by “Δ”, display the change in peak ΔF/F from ACSF to NBQX for each pixel, with white indicating no change, red indicating an increase, and blue indicating a decrease. These difference maps show that NBQX reduced responses to L4 stimulation (Fig. 6A, darkest blue under “Δ”) outside of the home barrel, while home barrel responses were unchanged or increased. Difference maps for stimulation in L2/3 showed that NBQX increased responses in L4 of both barrels over larger areas, and decreased responses in smaller areas (Fig. 6B).

We further analyzed this spatial variation in the action of NBQX by plotting difference histograms for pixels in the neighboring barrel (to the right of the difference maps). Neighbor barrel distributions had means significantly less than zero for L4 stimulation (one-tailed 1-sample t-test, L4, top: T = −8.7, p < 0.001, middle: T = −38, p < 0.001, bottom: T = −33, p < 0.001) but not for L2/3 stimulation (top: T = 15, p = 1.0, middle: T = 9.4, p = 1.0 , bottom: T = 6.1, p = 1.0). This analysis illustrates the extensive dependence on excitatory synapses of responses of neighboring barrels to L4 stimulation compared to the limited dependence with L2/3 stimulation. To evaluate the areas over which NBQX had an effect we implemented a slice-specific ΔF/F threshold (70^th^ percentile of recording pixels in ACSF) to calculate the number of responding pixels (36 μm^2^ per pixel). The area responding to L2/3 stimulation was not significantly different between NBQX and control (Welch’s t-test, T = 0.15, p = 0.88, n = 6 slices), while with L/4 stimulation NBQX produced a large and significant decrease in response area (T = 2.8, p = 0.018). Peak ΔF/F of neighbor barrels was also decreased significantly by NBQX for L4 stimulation (T = 3.1, p = 0.01), but the decrease was smaller and not significant for L2/3 stimulation (Fig. 6D, T = 0.08, p = 0.94).

The responses seen prior to NBQX addition were composed of direct responses, antidromic responses, and synaptic responses superimposed, and were often biphasic (as in Fig. 5C). NBQX removed the second component (Fig. 5D). This suggests that synaptic delays on the order of several milliseconds separate synaptic from direct or antidromic responses. To gain a quantitative representation of the loss of the second slower phase after addition of NBQX, we evaluated the changes in response half-widths. In neighboring barrels NBQX reduced half-widths more than two-fold for L2/3 stimulation from 14.6 ± 2.9 ms to 6.4 ± 0.8 ms (Fig. 6E; Welch’s t-test, L2/3 neighbor barrel: T = 2.7, p = 0.02). By contrast, the reduction for L4 stimulation from 15.2 ± 2.3 ms to 11.7 ± 4.7 ms was not significant (Fig. 6E; L4 neighbor barrel: T = 0.7, p = 0.5). This suggests that inhibitory circuitry is activated within the home barrel, and that it contributes to the termination of responses to stimulation of both L4 and L2/3.

### Propagation through layers and barrels

**Fig. 7:**
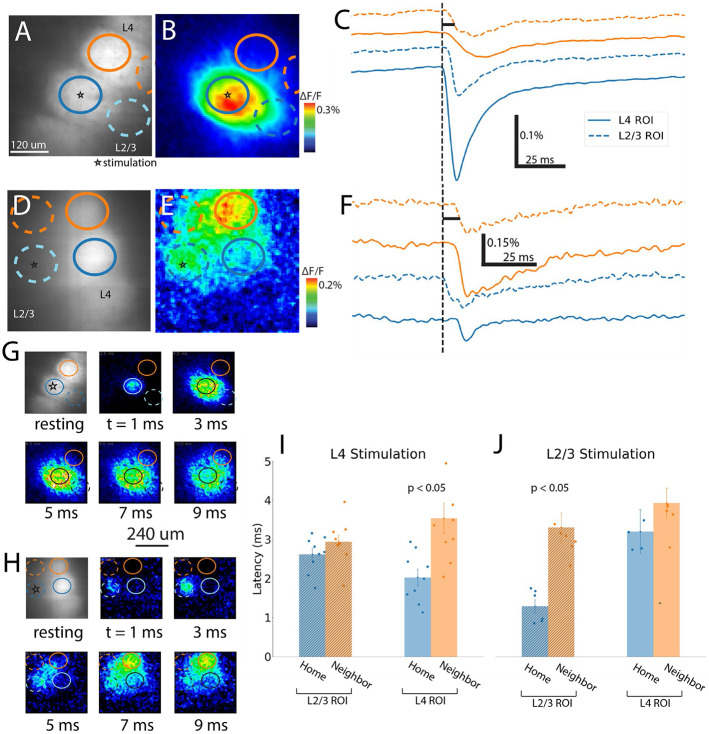
Spatiotemporal spread of excitation across barrels and layers. **A** and **D**. Fluorescence images showing ROIs and stimulation sites. **B** and **E.** Heat maps of peak responses (color scale denotes amplitude). **C** and **F.** Traces in the home (blue) and neighbor (orange) barrels of L4 (solid) and L2/3 (dotted). ROI outlines in maps correspond to traces with corresponding colors. Dotted black line marks stimulation time. With L4 stimulation, the response in the L4 home barrel precedes that in L2/3 and in neighbor barrels. **D-F.** Stimulation in L2/3 shows near-simultaneous activation of three nearby ROIs following activation of the stimulated L2/3 ROI. **G-H.** Upper-left: fluorescence images corresponding to A and D, with successive panels providing a time-lapse sequence of ΔF/F maps at 1 ms intervals following stimulation in L4 (G) or L2/3 (H) to illustrate the spread of excitation over a 9 ms window. **I-J.** Latency to half-maximum ΔF/F for each ROI averaged over multiple slices for L4 stimulation (n = 10) and L2/3 stimulation (n = 7). Means are plotted ± SEM with points for individual slices. L4 stimulation leads to sequential activation of L4 in home followed by neighbor barrels, while L2/3 stimulation leads to more synchronous activation across regions.

The dependence of inter-barrel L4→L4 communication on excitatory synaptic transmission through L2/3 suggests that response latencies should follow a sequence along the pathway of activity spread through this circuit. A fluorescence image of a slice shows the site of stimulation and the ROIs selected within L2/3 and L4 of the home and neighboring barrels (Fig. 7A). A map of peak amplitude demonstrates the spread of responses to L4 of an adjacent barrel (Fig. 7B). The traces in Fig. 7C reveal the time differences in response onset in different locations, and thus illustrate how depolarization spreads (vertical black dashed lines mark the stimulation time). Traces from L2/3 and L4 show that responses in L4 of the home barrel are followed first by responses in L2/3 of the home barrel, and then by responses in L4 in the neighboring barrel (Fig. 7C). We then stimulated in L2/3 in the slice shown in Fig. 7D to obtain a response heatmap (Fig. 7E) and traces (Fig. 7F). These traces illustrate that initial activation of L2/3 of the home barrel precedes activation of L4 of the home barrel, which is nearly simultaneous with activation of L4 and L2/3 in the neighboring barrel.

Sequences of amplitude maps at regular time intervals chart the spread of excitation evoked by stimulation of L4 (Fig. 7G) and L2/3 (Fig. 7H). These maps are snapshots in time, as opposed to peak response maps displayed in the preceding figures. Taking the latency as the time from stimulation to half-maximum on the rising phase (shown with solid black lines in the first traces of Figs. 7C and 7F), we find a latency of 1.9 ms for L4 home barrel responses to L4 stimulation), followed by L2/3 in the home barrels at 3.0 ms and in the neighbor barrel at 3.9 ms (Fig. 7G). The short latency in L4 of the home barrel reflects direct local conduction through axons and dendrites. L4 of the neighbor barrel is activated at the longest latency (6.2 ms), reflecting synaptic activation of L2/3 excitatory neurons, conduction through their axons, and synaptic excitation in L4. With L2/3 stimulation, local responses in L2/3 appear first (1.5 ms) followed within 3-4 ms by essentially simultaneous activation of the other three regions, starting in the 5-ms snapshot (Fig. 7H). Multimedia 1 presents another representative example (separate from Fig. 7G) of spread of responses to L4 stimulation, showing the evolution of a peak ΔF/F heatmap at the right with four traces, one for each barrel and layer, at the left. This animation illustrates the delay of L4→L4 spread and tracks the L4→L2/3→L4 conduction route. Multimedia 2 presents an example of L2/3 stimulation separate from the example in Fig. 7H, visualizing the simultaneous L2/3→L4 antidromic conduction and biphasic responses, here seen clearly in the L4 home barrel.

These sequences can also be seen in latency averages (Fig. 7I, n = 10). Stimulating L4 activated L4 of the home barrel (2.0 ± 0.2 ms), followed by L2/3 of the home and neighbor barrels (2.6 ± 0.2 ms and 2.9 ± 0.2 ms, respectively), and finally by L4 of the neighbor barrel (3.5 ± 0.4 ms). In contrast, stimulating L2/3 (Fig. 7J, n = 7) activated the local region very quickly (1.3 ± 0.2 ms) by exciting processes from L4 neurons. The L4 home and neighbor barrel responses followed nearly 2 ms later, with indistinguishable latencies (home: 3.2 ± 0.6 ms, neighbor 3.9 ± 0.4 ms). Activation of L2/3 of the neighbor barrel was also essentially coincident with activation of L4 (3.3 ± 0.4 ms), consistent with the stimulation site in L2/3 receiving projections from each of these three other regions (Fig. 4E). We found that the latency among each set of four groups differed significantly (ANOVA; Fig. 7I: F = 6.3, p = 0.0017; Fig. 7J: F = 7.9, p = 0.0010). In further support of communication through L2/3, the latency in L4 differed significantly between home and neighbor barrels with L4 stimulation (T = 3.4, p = 0.006) but not with L2/3 stimulation (T = 1.1, p = 0.32). For latencies of responses in L2/3 to L4 stimulation, there was no significant difference between home and neighbor barrels (T = 1.5, p = 0.17), as these sites fall midway along the L4→L2/3→L4 pathway. With L2/3 stimulation there was also no significant difference between home and neighbor barrel L2/3 response latencies (T = 4.6, p = 0.0026). In summary, these latencies in different locations to stimulation of different sites are all consistent with L2/3 serving as an intermediate relay for communication between adjacent L4 barrels (Fig. 4E).

### Intra- and Inter-barrel Velocity

**Fig. 8:**
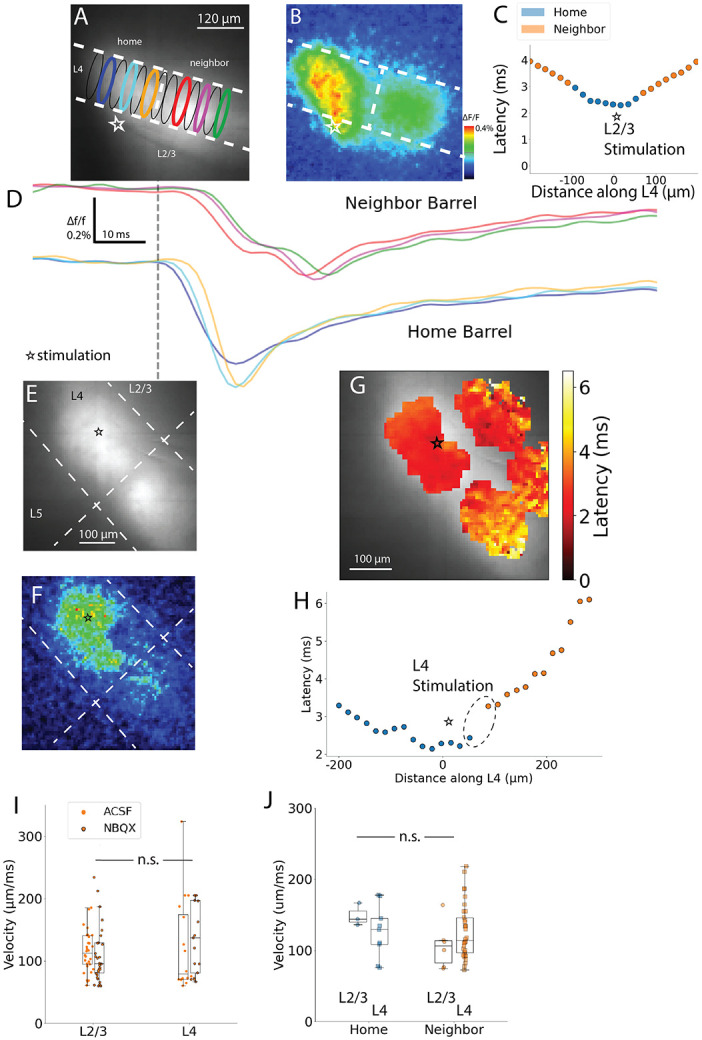
Velocity of propagation into neighbor barrels. **A**. Fluorescence image of a coronal slice showing ROIs used to compute velocity in home and neighbor barrels. **B**. Peak ΔF/F response map of the slice shown in A with stimulation site and layer/barrel boundaries annotated). **C.** Latency versus distance along L4 for responses to L2/3 stimulation, for a different example from the slice shown in A. Blue points are for the home barrel, while orange points are for neighbor barrels of either side. Domains of constant slope were analyzed separately for velocity measurements. **D**. ΔF/F traces from home and neighbor barrel ROIs of the corresponding colors in A arranged along the direction of propagation. **E.** Fluorescence image of a sagittal slice overlaid with latency map, with L4 stimulation site marked with a star. **F.** Peak ΔF/F response map with stimulation site and layer/barrel boundaries annotated for the slice shown in E. **G.** Heatmap of latency for the slice in E, laid over the resting fluorescence image. Latencies are shown only within the barrel ROIs, where fluorescence and thus responses are concentrated. **H**. ROIs from L4 in the example in E-G were used to plot latency versus distance along L4. The home and neighbor barrels show discontinuities in latency at the septum (black dotted ellipse). I. Boxplot and scatter of velocity measurements before and after NBQX. NBQX had no significant effect. **J.** Velocity was significantly greater within the home barrel than between barrels. Boxplots show minimum, first quartile, median, third quartile, and maximum with scatter; means ± SEM reported in Results. Statistics used barrel means as independent samples, although individual ROI measurements are displayed.

Response latencies generally increase with distance along pathways traced by axons, and the relation between latency and distance can be used to estimate conduction velocity. We constructed narrow ROIs 18-30 μm wide within L4 to measure intralaminar conduction velocity within and between barrels (30 μm wide ROIs are indicated in Fig. 8A). The heatmap in Fig. 8B shows responses to L2/3 stimulation that spread to L4 of the neighboring barrel. Plotting latency versus distance for the ROIs in L4, we find smooth propagation of responses to L2/3 stimulation, as illustrated in Fig. 8C. Traces from the ROIs in Fig. 8a are displayed in Fig. 8D for the home and neighbor barrels.

To visualize the propagation through the L4→L2/3→L4 pathway we stimulated in L4 (Fig. 8E-H). Resting fluorescence (Fig. 8E) and peak ΔF/F heatmap (Fig. 8F) are shown for a sagittal slice stimulated in L4. Fig. 8G displays a pixel-wise map of latency overlaid on the fluorescent image. The latencies are short in the home barrel (upper left barrel), and the increase toward L2/3 illustrates propagation in that direction. Further increases along L2/3 (right) illustrate propagation into the neighbor barrel (Fig. 8G), following the sequence found in Fig. 7G. Further increases in latencies along the neighbor barrel (lower left) illustrate propagation from L2/3→L4, with much of L4 in the neighboring barrel activated last.

In Fig. 8H, the ROIs along L4 (orange) show a jump of 0.60 ms over a distance of 18 μm (within black dotted ellipse), compared to the mean difference of 0.21 ± 0.04 ms per 18 μm in this plot. Such jumps were not seen for responses to L2/3 stimulation (Fig. 8C). One L4 ROI within the septum is excluded to avoid over-extrapolating latency in dim-fluorescence ROIs. However, other slices show accelerations rather than delays in their L4 trajectories, or have a delay that occurs well into the neighboring barrel. These qualitative boundary changes in velocity mark the transition between direct activation of the L4 home barrel to indirect activation of the neighboring barrel through L2/3. The heterogeneity of arrival times of responses in the neighboring barrel may reflect multiple parallel paths of inter-barrel communication, including mono- or multi-synaptic parallel pathways through L2/3, or feedforward signaling within L4 of the neighboring barrel (Fig. 8C).

Velocities were averaged over barrels for propagation within L4 of the neighbor barrel. The intra-barrel velocity was similar regardless of stimulation site (Fig. 8I, open circle markers; L2/3: 129 ± 12 μm/ms, L4: 122 ± 5 μm/ms, T = 0.98, p = 0.36). We also analyzed velocities in the presence of NBQX and observed no significant differences (Fig. 8I, bordered circle markers; ACSF and NBQX, L2/3: T = 1.16, p = 0.27, L4: T = 1.0, p = 0.34). This suggests that antidromic conduction determines the velocities of responses to L2/3 stimulation, despite the biphasic nature of these responses in neighboring barrels (Fig. 8D, top traces). L4 neighbor velocities were computed for the minority of cases where NBQX did not completely abolish responses (ACSF: n = 31 responding L4 neighbor barrels, NBQX: n = 6). Velocities were greater in the home barrel (132 ± 10 μm/ms) than the neighboring barrel with both L2/3 or L4 stimulation (mean: 123 ± 6 μm/ms), but this difference was not significant (T = 0.77, p = 0.45, Fig. 8J).

### Spatiotemporal biases in inter-barrel communication

Natural sensory input generally activates whiskers in characteristic sequences along rows and arcs to activate barrels in corresponding sequences. Directional biases in inter-barrel communication will determine how the BC integrates and processes this input. Coronal (Fig. 1A-B) and sagittal (Fig. 1C-D) slices differ in their inter-barrel relations. Barrels in sagittal slices track rows and arcs that map onto the rostro-ventral whisking direction toward which foveal protraction occurs, while barrels in coronal slices track along rows and arcs mapping along the rostro-dorsal whisking direction of exploratory protraction. Successive whisker contacts occur several milliseconds apart moving opposite to the direction of whisking. To study multi-barrel processing during whisking, we grouped our data by direction, plane, aspect, and phase (protraction versus retraction) to determine how inter-barrel signaling varies in response half-width, response latency, and neighbor/home ΔF/F ratio, as well as within-barrel velocity.

**Fig. 9.**
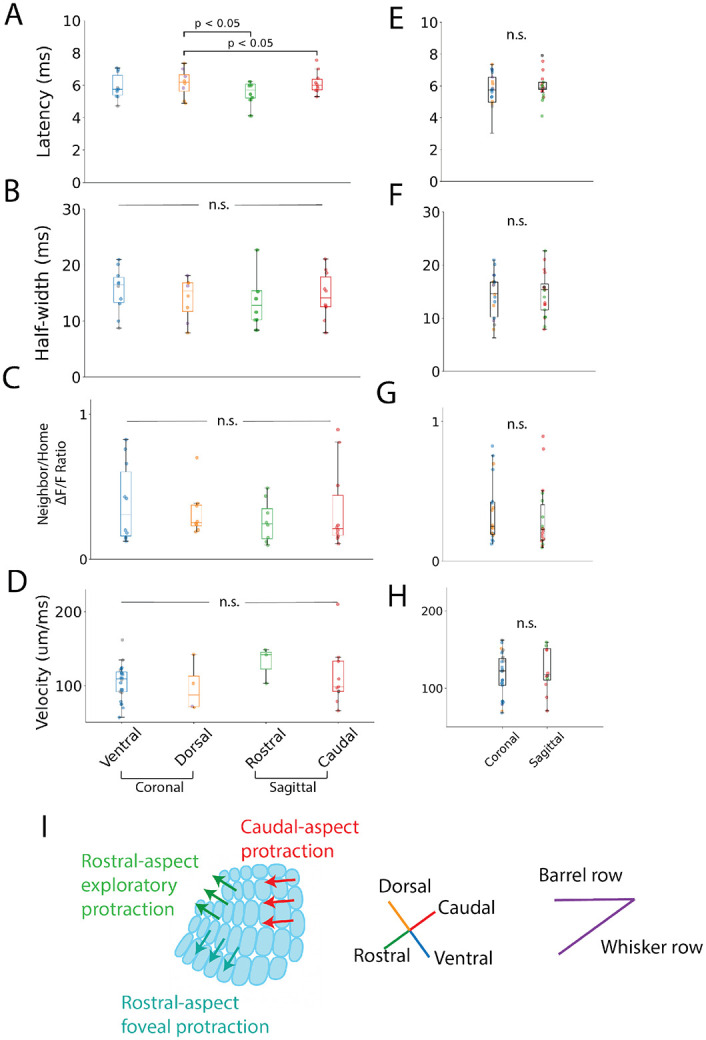
Comparison of responses between directions in barrel field. Latency (**A, E**), half-width (**B, F**), neighbor/home ΔF/F ratio (**C, G**) and velocity (**D, H**) of neighbor barrel responses grouped by propagation direction: rostral, caudal, dorsal, or ventral relative to the home barrel (A-D) and grouped by slice plane: coronal or sagittal (E-H). Colors denote Caudal in M-S slices, Rostral in M-S slices, Dorsal in C-C slices, Ventral in C-C slices, Dorsal in R-C slices, and Ventral in R-C slices; R-C and C-C of the same direction are grouped in boxplots and scatter plots. Boxplots show minimum, first quartile, median, third quartile, and maximum with scatter; means ± SEM reported in Results. Statistical analysis used one-way ANOVA or Welch’s t-tests as appropriate. **I**. Diagram relating the kinematics of whisking patterns to barrel layout, showing that the dorsal-ventral slice plane in coronal slices tends to align along exploratory whisking, and the rostral-caudal slice plane in sagittal slices aligns along foveal whisking (Knutsen et al., 2005; Petersen et al., 2020). Orientation of four directions is based on the Brain Globe 3D atlas of barrel cortex viewed in coronal and sagittal planes (Claudi et al., 2020).

Fig. 9 plots barrel-averaged latency, half-width, neighbor/home ΔF/F ratio, and within-barrel conduction velocity grouped into four directions, with colors representing aspect-specific directions. Figs. 9A-D present values grouped by direction in the barrel field, and Figs. 9E-9H present values grouped as coronal (R-C and C-C) and sagittal (M-S) to investigate differences due to section plane alone. We compared four direction groups (lumping R-C and C-C together) and two plane groups (additionally lumping non-orthogonal directions together). We also compared protraction-related directions (ventral, or exploratory protraction, and caudal, or foveal protraction) to retraction-related directions (dorsal, or exploratory retraction, and rostral, or foveal retraction) using the sum contrast model described in Methods.

Mean response latencies measured here ranged from 5.8 to 6.2 ms. These values are on par with the time between successive whisker contacts, and are thus relevant to inter-barrel communication during multi-whisker deflection. Latencies of neighbor barrels varied between the four directions (caudal: 6.2 ± 0.2 ms; rostral: 5.8 ± 0.4 ms; dorsal: 6.1 ± 0.3 ms; ventral: 5.9 ± 0.3 ms; mean effect size for contrast model: 0.23), and the differences were significant (Fig. 9a; ANOVA, F = 5.9, p = 0.001, n = 10, 8, 8, 10 slices), with caudal > dorsal (Tukey’s HSD: p = 0.001) and dorsal > rostral (p = 0.001). Comparisons showed no significant differences (Tukey’s HSD; p = 0.9, 0.11, 0.073, 0.12) between planes (Fig. 9E, Welch’s t-test, T = 1.7, p = 0.11) and between protraction and retraction phases (T = 0.63, p(32) = 0.27).

We then examined mean half-widths of neighbor barrel responses in four directions in the barrel field (Fig. 9B; caudal: 14.6 ± 1.3 ms; rostral: 13.5 ± 1.6 ms; dorsal: 14.0 ± 1.3 ms; ventral: 15.5 ± 1.3 ms; mean effect size (contrast model): 0.65). These values were indistinguishable across direction groups (ANOVA, F = 0.5, p = 0.77, n = 10, 8, 8, 10) and between coronal and sagittal slice planes (Welch’s t-test, T = 0.75, p = 0.46). The contrast model showed no significant difference in half-width between protraction and retraction directions (T = 1.0, p(32) = 0.16).In light of the known anisotropy in barrel fields (Hubatz et al., 2020; Petersen et al., 2003; Vilarchao et al., 2018), this isotropy suggests directional biases arise from extracortical sources (such as thalamic or thalamocortical processing), or from other intracortical sources such as short-term plasticity.

Rather than compare ΔF/F of neighbor barrels between slices, we normalized each neighbor barrel ΔF/F to the home barrel ΔF/F of that slice. This accounted for slice-to-slice variation (Fig. 9C; caudal: 0.35 ± 0.09; rostral: 0.26 ± 0.05; dorsal: 0.32 ± 0.06; ventral: 0.39 ± 0.09; Fig. 9G; coronal: 0.36 ± 0.05; sagittal: 0.31 ± 0.05; mean effect size (contrast model): 0.24). Direction did not have a significant effect on neighbor/home ΔF/F ratio (ANOVA, F = 0.45, p = 0.72), nor did slice plane (Welch’s t-test, T = 0.67, p = 0.51) or phase (sum contrast model, T = 1.0, p(32) = 0.15). This isotropy implied that anisotropy in paired-pulse experiments (presented below) was a result of differences in ΔF/F of the second pulse.

Fig. 9D compares propagation velocity entirely within L4 of neighboring barrels in response to L4 stimulation, focusing on within-barrel speed as distinct from inter-barrel communication latency. Direction did not have a significant impact on velocity (ANOVA, F = 0.69, p = 0.56; ventral: 104 ± 5 μm/ms, n = 19; caudal: 113 ± 14 μm/ms, n = 9, dorsal: 97 ± 17 μm/ms, n = 4; rostral: 131 ± 14 μm/ms, n = 3; mean effect size of contrast model: 1.25). Grouped by slice plane the smaller mean coronal velocity (117 ± 5 μm/ms, n = 29) was not significantly different from the mean sagittal velocity of 133 ± 14 μm/ms, n = 12 (Fig. 9H; T = 1.1, p = 0.30). We had hypothesized that sagittal velocities would be faster than coronal; whisking velocity is greater along the rostral-caudal axis (Petersen et al., 2020), requiring faster intra-barrel processing to preserve temporal fidelity as whiskers sweep greater angles. Additionally, the larger barrels in the caudal aspect of BC may also require faster conduction to communicate within a greater volume. With its more specific test, the contrast model showed that protraction velocities were not significantly smaller than retraction velocities, consistent with the similar latencies (T = 0.12, p(36) = 0.45); Fig. 9I displays the barrel organization to help relate the directions between barrels in slicing planes to the angular offset of whisking field by showing the whisking phase relevant to each BC slice plane. Conduction velocity, half-width, and ΔF/F in response to single-pulse stimulation appear isotropic across the barrel field, while differences in inter-barrel latency were significant.

### Paired-pulse depression

**Fig. 10:**
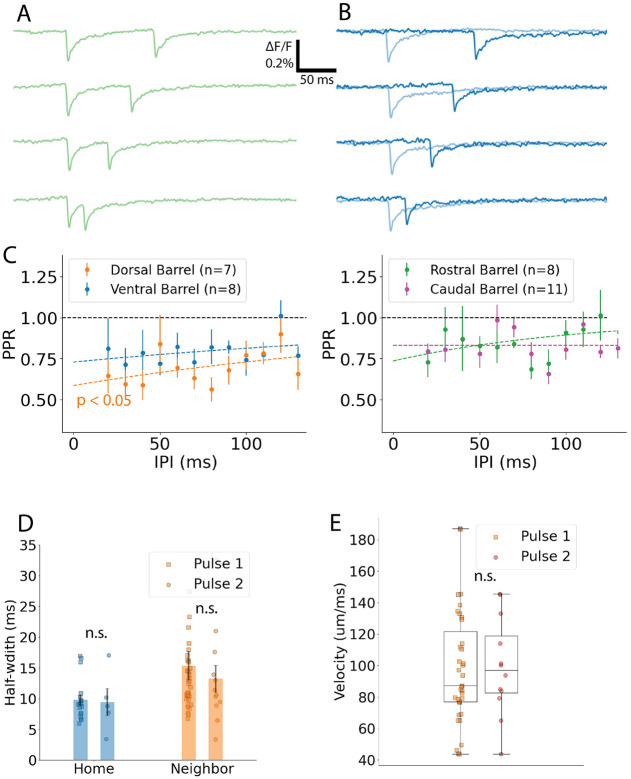
Paired-pulse ratio in neighbor barrels across directions in barrel field. **A** Fluorescence traces from barrel delimited ROIs in L4 for paired-pulse stimulation at four different inter-pulse intervals (IPI). At sufficiently small IPIs, the first response does not return to baseline, leading to temporal summation that increases the second response. **B**. Single-pulse control traces are recorded (light blue traces) immediately before or after (determined randomly) the paired-pulse traces. Subtracted traces (dark blue traces), computed as paired-pulse minus single-pulse, remove temporal summation. **C-D.** Paired-pulse ratio (PPR, the amplitude of the second response divided by the first) plotted as a function of IPI for each direction, for coronal (C) and sagittal (D) slices. Solid curves represent exponential fits to recovery (see text). **D.** No significant change in velocity between the first and second responses. **E.** Response half-width was also unchanged. Note that the difference between neighbor and home half-widths was discussed in regard to Fig. 3H, so we have left it unmarked here to avoid repetition of results and to focus on the present comparison of first and second responses. Multimedia 1: Spatiotemporal spread of excitation across barrels and layers for L4 stimulation. Left: Animated sequence of heat maps of peak responses from just before stimulation to 10 ms post-stimulation, paralleling the heatmap time series of Fig. 7G for a separate example. The resting fluorescence image is shown at the animation end. Stimulation site is marked with a star. Right: Traces in the home (L4: blue, L2/3 green) and neighbor (L4: orange, L2/3: red) barrels. ROI outlines of the same color in maps correspond to traces. Dotted blue line marks stimulation time. With L4 stimulation, the response in L4 of the home barrel precedes that in L2/3 and in neighbor barrels. Multimedia 2: Stimulation in L2/3 shows near-simultaneous activation of three unstimulated ROIs. Left: Heat maps of peak responses animated from just before stimulation to 10 ms post-stimulation, paralleling the heatmap time series of Fig. 7H for a separate example. The resting fluorescence image is shown at the animation end. Stimulation site is marked with a star. Right: Traces in the home (L4: blue, L2/3 green) and neighbor (L4: orange, L2/3: red) barrels. ROI outlines of the same color in maps corresponding to traces. Dotted blue line marks stimulation time. Biphasic responses reflect multiple L2/3-L4 pathways mediating inter-barrel communication.

Whisking elicits repetitive activity at characteristic frequencies, and short-term synaptic plasticity will contribute to the processing of these inputs. Since our results indicated that L4→L4 communication depends on excitatory synaptic transmission, we investigated short-term plasticity of this pathway. We stimulated L4 with pairs of pulses and evaluated the ratio of responses in L4 of neighboring barrels. Temporal summation was evident for inter-pulse intervals less than 80 ms (Fig. 10A) so we subtracted single-pulse controls from the paired-pulse responses (dark blue traces in Fig. 10B). The subtracted traces reveal paired-pulse depression and a slow recovery over IPIs from 20-120 ms. Figs. 10C-D plot paired-pulse ratio (PPR) versus IPI for neighboring barrel responses in the four different directions in the barrel field. IPI had a significant effect on PPR across groups (2-way ANOVA, PPR ~ IPI + C(Direction) + IPI:C(Direction), F = 5.3, p = 0.021), and the interaction between IPI and direction was also significant (F = 3.2, p = 0.012). This IPI-direction interaction was also significant between ventral and dorsal directions (2-way ANOVA; F = 8.8, F = 0.0034) but not between rostral and caudal directions (F = 0.42, p = 0.52). Other variables did not have a significant effect by themselves (ventral/dorsal, direction: F = 2.9, p = 0.089; IPI: F = 2.5, p = 0.12; rostral/caudal, direction: F = 0.17, p = 0.69; IPI: F = 0.30, p = 0.59). We fitted these PPR versus IPI plots to the exponential function $PPR=1-ae^{-\frac{IPI}{\tau }}$ to determine the recovery time constant, τ and PPR intercept at IPI = 0 ms, or 1 – a. The qualities of fit were modest for three of four directions (dorsal R^2^ = 0.20, ventral R^2^ = 0.14, rostral R^2^ = 0.28, caudal R^2^ = 0.002), suggesting additional dynamics beyond a single-exponential recovery from vesicle depletion, with possible contributions from facilitation, receptor desensitization, or calcium-sensing proteins (Chiu & Carter, 2024; Regehr, 2012).

Table 1 summarizes the 0 ms PPR-intercept and recovery time constant. The mean effect size among these groups is 0.84 (contrast model). All four directions show significant depression, with the PPR intercept significantly less than 1 (Dorsal: T = 5.6, p = 0.0006, ventral: T = 5.4, p = 0.0009, caudal: T = 3.2, p = 0.005, rostral: T = 3.1, p = 0.009). Among the groups, the PPR intercept varies significantly (ANOVA, F = 8.7, p = 0.0003). The dorsal PPR intercept is significantly less than those of the other three groups (Tukey’s HSD: caudal > dorsal, p = 0.001; rostral > dorsal, p = 0.032; ventral > dorsal, p = 0.025). Notably, the protraction PPR intercept was significantly greater than that of retraction (sum contrast-coded linear regression one-sided t-test, T = 6.3, p(30) < 0.001). Specifically, this reveals that ventral neighbor PPR is greater than dorsal neighbor PPR in foveal whisking, and caudal greater than rostral in exploratory whisking, resulting in a consistently greater protraction PPR. During the protraction phase of free whisking this weaker paired-pulse depression in the caudal direction may preserve temporal fidelity for input moving along this specific direction.

Direction also had a significant impact on the recovery time constant (ANOVA, F = 8.2, p = 0.002). Dorsal and ventral barrel recovery kinetics are not significantly different (Tukey’s HSD: p = 0.9). Although the ventral and dorsal recovery time constants were twice the rostral value, this difference was not significant (rostral versus ventral, p = 0.053; dorsal versus rostral, p = 0.15). In contrast, as suggested by the low quality of fit, caudal barrels do not show recovery within this range of IPIs, and show the least depression among all the directions (Fig. 10C).

Depression in amplitude was not accompanied by a change in half-width (Fig. 10D, home: T = 0.14, p = 0.89; neighbor: T = 0.66, p = 0.51). Our NBQX results suggest interneuron recruitment is relevant on this time scale, enabling feedforward inhibition to limit response durations. Thus, a constant half-width from the first to second pulse of a pair appears consistent with previous studies of paired-pulse depression preserving excitation/inhibition balance (Chelaru & Dragoi, 2007; Lombardi et al., 2023). Additionally, there was no change in conduction velocity from the first to second pulse (Fig. 10E, 99 ± 9 μm/ms to 98 ± 8 μm/ms, T = 0.11, p = 0.91), suggesting a constant speed for processing information between adjacent barrels.

## Discussion

We imaged evoked voltage changes in Scnn1a-expressing excitatory neurons in L4 of mouse BC slices to characterize inter-barrel communication. Varying the stimulation site revealed distinct propagation patterns underlying inter-barrel signaling. Neurons in L2/3 relayed L4 excitation between adjacent barrels. Inter-barrel latency was longest in the dorsal direction, but neighbor barrel conduction velocity, half-width, and amplitude ratio were found to be isotropic. Paired pulse depression was asymmetric, with greater depression in the retraction direction. These findings reveal direction-, frequency-, cell type- and layer-specific mechanisms shaping somatotopic information flow in the BC microcircuit, refining our understanding of cortical sensory processing.

### Mechanistic insights into inter-barrel signaling

L4 stimulation elicited AMPA receptor-dependent responses in L4 of neighboring barrels. In contrast, L2/3 stimulation elicited AMPA receptor-independent responses in L4 of both home and neighboring barrels. This pattern is consistent with antidromic activation of L4 pyramidal neurons via the extensive inter-barrel arborization in L2/3 (Feldmeyer et al., 2002; Staiger & Petersen, 2021) as well as previous studies noting the functional independence of barrels in L4 (Hill & Greenfield, 2014; Laaris & Keller, 2002; Petersen & Sakmann, 2001; Sato et al., 2008). This L4→L/23→L4 pathway can contribute to intra-barrel processing of thalamic input to L4. NBQX not only blocked neighboring barrel responses to L4 stimulation but also narrowed response half-widths, resolving direct from synaptic responses. NBQX also revealed an inhibitory action: blocking excitatory transmission increased depolarization, suggesting that AMPA receptor-mediated activation of inhibitory neurons engages feedforward inhibition within and between barrels, to limit response amplitude on the timescale of several milliseconds. There could be multiple parallel pathways between L4 of different barrels, the likely candidates being $L4\to L2∕3^{home}\to L2∕3^{neighbor}\to L4$; and $L4\to L2∕3^{home}\to L4$; with lesser contributions from $L4\to L2∕3^{neighbor}\to L4$ (where $L4\to L2∕3^{neighbor}$ may be either antidromic or via less-common L4 pyramidal neuron projections that cross into L2/3 of an adjacent barrel). L5 may also contribute, but we positioned slices to include L4 and L2/3 at the expense of L5, and did not study responses in this layer systematically.

### Comparison to previous intracortical velocity studies

Our velocities (104-131 μm/ms) were generally faster than previous *in-vivo* measurements. Responses to single-whisker deflections spread at 60 μm/ms along rows and 33 μm/ms along arcs in L2/3 (Petersen et al., 2003), ranging from 35 μm/ms (spontaneous (Reyes-Puerta et al., 2016)), to 50 μm/ms (Armstrong-James et al., 1992), to 95 μm/ms (multi-whisker deflection (Vilarchao et al., 2018)). However, a velocity of 200 μm/ms has been reported (Lippert et al., 2007). Slice measurements of BC conduction velocity are similar to those reported here: 74-473 μm/ms (Scheuer et al., 2023), 160 μm/ms (Haupt, 2000), and 130 μm/ms (Laaris & Keller, 2002). Naturalistic stimuli may recruit cortical neurons more gradually via parallel corticothalamic inputs (Ma & Patel, 2021), while the direct electrical stimulation of slices in the present study induces an abrupt and synchronous depolarization in many local neural elements. While natural responses or spontaneous activity may reflect system-level function, state-dependent behavior, and longer-range connections of the circuit, electrical stimulation in vitro reveals the underlying intracortical axonal conduction velocities and synaptic delays of specific microcircuit components. Moreover, repetitive electrical stimulation (100-500 Hz) leaves in-vivo velocities unaffected (113 μm/ms (Margalit & Slovin, 2018)). Likewise, velocity is unchanged during repetitive free whisking (Ferezou et al., 2006), consistent with our observation of constant velocities between successive pairs of pulses.

### Directional anisotropy and propagation dynamics

Our experiments probed response duration, ΔF/F ratios, latency, intra-barrel velocity, and PPR of neighboring barrels for directional preference. We interpret inter-barrel anisotropies through three normative perception models: dorsal-ventral versus rostral-caudal whisking axes, protraction versus retraction, and rostral versus caudal whisker fields. Anisotropy further divides into fast foveal (with rostro-ventral protraction) versus slow exploratory (with rostro-dorsal protraction) behavior. Differences in barrel and whisker dimensions also shape asymmetric processing.

While half-widths, ΔF/F ratios, and conduction velocities of neighbor barrel responses were isotropic in all directions, latencies were longest in the caudal direction. Whisker contact with an object is communicated to its caudal neighbor barrel approximately 10 ms after the adjacent caudal whisker contacts the same object (Vilarchao et al., 2018). In particular, this asymmetry parallels the lower density of barrel projections from L4 neurons to L2/3 caudal neighbors (Bernardo et al., 1990), as the shortest axonal path will play an outsized role in determining latency, and a larger sample of paths will decrease the length of the shortest path, by Fisher-Tippett-Gnedenko theorem (Fisher & Tippett, 1928). The neighbor barrel response is relevant to integration of a subsequent whisker contact: caudal latencies relate to inter-barrel integration during protraction, and dorsal latencies relate to exploratory integration during retraction. However, single pulse stimulation mimics single-whisker deflection better than the steady state whisking during which these protraction and retraction phases become relevant. Thus, the barrel location and role within the whisking field may be more relevant to its single-pulse stimulation response than whisking direction or phase.

Several factors may shape responses to single-pulse stimulation. Barrels located in the caudal BC (e.g. in M-S slices) sense peripheral areas of the receptive field, enabling faster processing of strong peripheral stimuli during rest, which may require an immediate shift in attention. Barrel size differences may also shape velocities—rostral barrels were 17% smaller than caudal barrels (Fig. 2D-E), enabling barrel-wide communication with 14% (not significant) slower velocities. Larger barrels, corresponding to the longest and thickest whiskers, receive stronger excitation (Grant et al., 2009; Woolsey, 1970). Since speed increases with signal amplitude across cortical areas (Afrashteh et al., 2021), these highly-excited larger barrels may specialize in faster barrel-wide propagation. Long, caudal whiskers may specialize in slow, exploratory whisking compared to the small, rostral whiskers preferentially recruited during foveal whisking (Hobbs et al., 2016).

### Anisotropic short-term depression

Paired-pulse stimulation allowed us to study inter-barrel STP relevant to repetitive whisking, and revealed direction-dependent variations in short-term plasticity: retraction-directed responses showed the strongest depression at short IPIs, and protraction-directed responses, especially caudal, responses showed the least depression. While protraction is primarily performed in the rostral direction, protraction also extends ventrally when the whiskers are near their rostral-most setpoint (Petersen et al., 2020), where a mouse whisks while making contact with a vertical pole. This implies a similar relationship between a whisker and successive dorsal contacts. However, protraction in free air produces a dorsal rather than ventral change in whisker elevation (Knutsen et al., 2005), reflecting the behavioral difference between wide-angle exploratory whisking before contacting objects, and foveal whisking fixated on a precise location. The higher PPR in protraction—especially caudal—directions will make transmission of repetitive activity more reliable in these directions.

IPIs of 20-100 ms are relevant to repetitive thalamocortical input to the same whisker, falling into the 10-50 Hz range that is sub-linearly boosted during thalamic processing in the 5-25 Hz whisking range (Sosnik et al., 2001). The repeated thalamocortical input to an L4 barrel propagates to neighboring barrels, during which depression of communication with neighboring barrels becomes relevant. At steady state, inter-barrel communication is attenuated more strongly in the direction of retraction, balancing the high excitation and preserving the fidelity of protraction phase information. This asymmetry enhances multi-barrel integration during the information-rich protraction which has a 30% longer duration than retraction (Bermejo et al., 2002; Leiser & Moxon, 2007). The shorter phase of retraction results in more synchronous excitatory input, which is more strongly attenuated in preparation for the next protraction. This excitatory processing cycle provides a synaptic circuit explanation for how unequal protraction and retraction durations may enhance sensory processing.

## Conclusion

Combining voltage imaging in genetically-defined neurons with slice alignment to the BC anatomy enabled us to connect population-level responses to system-level behavior. Inter-barrel communication between L4 excitatory neurons depends on layer-specific pathways that are modulated to degrees that depend on direction and frequency. The L4→L2/3→L4 circuit supports inter-barrel signaling, and its anisotropy in latency and synaptic depression can serve to tune intracortical communication to the spatiotemporal structure of whisker input. These mechanisms serve in the interpretation of whisking phases, whisker shape and location, whisking behavior type, and frequency. More broadly, this study suggests that spatiotemporal alignment to sensory input may be relevant to how microcircuits implement cortical somatotopic maps in general. Extending this investigation of inter-barrel communication to the single-cell level with sparse CreERT2-driven labeling (Feil et al., 2009) and single-cell stimulation (Canales et al., 2022) has the potential to reveal the detailed circuitry underlying the processing of sensory inputs.

## Supplementary Material

Supplement 1

Supplement 2

## Figures and Tables

**Table 1.** The PPR intercept at 0 ms and the exponential recovery time constant τ for each direction of inter-barrel propagation, determined by fitting the equation in the text. The caudal direction did not show measurable recovery within the 120 ms IPI testing range, so τ was not determined. Values are presented as mean ± SEM (n = number of slices).

	PPR intercept, 1-a	recovery time constant τ (ms)	n
Dorsal	0.58 ± 0.08	235 ± 150	7
Ventral	0.73 ± 0.06	269 ± 230	8
Caudal	0.83 ± 0.06	ND	8
Rostral	0.73 ± 0.12	110 ± 85	11

## Significance

The barrel cortex processes tactile information from whiskers with high spatial and temporal precision. Using cell-type-specific voltage imaging in anatomically aligned slices, we examined how excitatory neurons communicate between neighboring barrels. We identified a layer-specific relay between barrels and found that response latency and short-term synaptic depression vary with propagation direction. This anisotropy is tailored for processing the kinematics of natural whisker motion, and suggests that local microcircuits are tuned to preserve temporal fidelity during slow, focused whisking, and to filter redundant inputs during rapid, exploratory phases. By linking synaptic properties, circuit connectivity, and natural behavior, this work advances our understanding of how neocortical networks process inputs and support active sensing.
