# Supplementary material for: Biased inter-columnar communication and short-term plasticity in mouse barrel cortex
Source: bioRxiv. 2026 Jan 12:2025.11.04.686617. Originally published 2025 Nov 6. Preprint. [Version 2] doi: 10.1101/2025.11.04.686617 (PMC12637616; doi:10.1101/2025.11.04.686617)
Supplement: Supplementary file 1 [file NIHPP2025.11.04.686617v2-supplement-1.pdf]

bioRxiv preprint doi: https://doi.org/10.1101/2025.11.04.686617; this version posted January 12, 2026. The copyright holder for this preprint (which was not certified by peer review) is the author/funder, who has granted bioRxiv a license to display the preprint in perpetuity. It is made available under a CC-BY 4.0 International license.

Supplementary information for:
Biased inter-columnar communication and short-term plasticity in mouse barrel cortex

John M. Judge[1]
Meyer B. Jackson[1,2]

jjudge3@wisc.edu
mbjackso@wisc.edu

[1]Biophysics PhD Program,
[2]Dept. of Neuroscience
U. of Wisconsin-Madison
Wisconsin Institutes for Medical Research
1111 Highland Ave
Madison, WI 53705

bioRxiv preprint doi: https://doi.org/10.1101/2025.11.04.686617; this version posted January 12, 2026. The copyright holder for this preprint (which was not certified by peer review) is the author/funder, who has granted bioRxiv a license to display the preprint in perpetuity. It is made available under a CC-BY 4.0 International license.

Supplementary Methods

Analysis, Statistics. PhotoZ implements baseline subtraction, temporal and spatial filtering, and linearly-interpolated measurements of latency, half-width, and peak ΔF/F measurements. The latencies are times from the stimulation pulse to half-amplitude. Time windows for analysis were from 3 ms before stimulation to 50 ms post-stimulation, with independent samples obtained as barrel-averaged measurements. Domains with lengths of at least 50 μm showing constant velocity were identified for linear regression and velocity measurement. Velocity was calculated with the SciPy.stats Python library with linear regression on latency versus distance along L4, using 7 or more regions of interest (ROIs) in each barrel. Pixels within 50 μm of the stimulation site were excluded. Septa between barrels were also excluded due to their dim fluorescence.

We tested multiple groups for significance with one-way or two-way ANOVA (SciPy.stats). If ANOVA indicated significant variation, Shapiro-Wilk tests confirmed normality before we used Tukey's honestly significance difference (HSD) test for pairwise comparisons (Statsmodels, Python). We confirmed that inter-class variance was not significantly greater than intra-class variance with a mixed-effect linear regression model implemented in R, obviating the need to control for pseudoreplication effects between slices of the same animal or litter (Lazic, 2010). In cases where only two groups were compared, we used one-sided Welch's t-tests (Scipy.stats). We also used one-sample one-sided t-tests (Scipy.stats) to compare mean against null hypothesis values where relevant.

To test the dependence of measurements on direction we hypothesized that the differences between dorsal-minus-ventral and rostral-minus-caudal will share the same sign because ventral and caudal neighbor activations are associated with whisker protraction, whereas dorsal and rostral neighbor activations are associated with retraction, taking the angular offset between BC and whisker field into account. Because each group is comprised of independently measured barrels, we used a contrast coding system of linear regression (Patsy Python:sum contrast coding; Statsmodel's ordinary least squares function). Using this sum contrast coding (comparing to the group mean as reference), we tested whether the differences of each pair of group means (represented by μ_i) would sum to significantly greater than zero on average (one-sided t-test), i.e. alternative hypotheses of:

$$(\mu_{dorsal} - \mu_{ventral}) + (\mu_{rostral} - \mu_{caudal}) > 0$$

This tested for protraction < retraction; signs were flipped to test for protraction > retraction. The contrast model allowed for specific testing without the family-wise error rate corrections in Tukey's HSD test.

We designed our sample sizes to detect intracortical processing differences on the order of at least 15%. To achieve a standard power level (0.8) and false positive rate of 5%, allowing for an uncertainty of 8%, we calculated that a minimum sample size of 8 is needed: $n_2 = n_1 = \frac{\sigma_1^2 + \sigma_2^2/K}{\Delta^2}\left(z_{1-\alpha/2} + z_{1-\beta/2}\right)^2 = 4$. For post-hoc power analysis, we measured variances (ΔF/F ratios: 14%, half-width: 25%, velocity: 19-26%, latency: 10-19%, paired-pule ratio (PPR) parameters: 20-50%) and calculated effect sizes and sample sizes based on the contrast model (reported in Results), which are similar to those of the sagittal-coronal comparison. Tests of half-width (0.15), ΔF/F ratios (0.17), and latency (0.54) were underpowered. The power levels of velocity (1.0) and PPR at 20 ms interpulse interval (IPI) (0.73) indicated a sufficient sample size to achieve standard statistical power.

bioRxiv preprint doi: https://doi.org/10.1101/2025.11.04.686617; this version posted January 12, 2026. The copyright holder for this preprint (which was not certified by peer review) is the author/funder, who has granted bioRxiv a license to display the preprint in perpetuity. It is made available under a CC-BY 4.0 International license.

Supplementary Results

Our supplementary results include further details on barrel-to-whisker alignment, an analysis of barrel dimensions of each type of slice, a summary of non-significant results for response properties grouped by direction, and results demonstrating controlled variables do not have unexpected effects on the velocity and PPR results.

As illustrated in Fig. 1, we categorized our slices by their preservation of connections. The connections are preserved depending not only on the plane of section but also on slice origin (e.g. coronal sections from rostral versus caudal aspects, as in Fig. 1A). This produces the four categories specified in Results: rostral-aspect coronal (R-C slice, Fig. 1A, 1B), caudal-aspect coronal (C-C slice, Fig. 1A, 1C), lateral-aspect sagittal (L-S slice, Fig. 1D, 1E), and medial-aspect sagittal (M-S slice, Fig. 1D, 1F). R-C slices tend to present a cross-section of rows C, D, or E (labeled in Fig. 1B, bottom right), which curve into the rostro-dorsal direction in the rostral aspect. (Fig. 1A, right). These rows have narrow septa and higher barrel count (7-10 per slice). In contrast, C-C slices present fewer, larger barrels and septa (Fig. 1C). Sagittal slices originated from either aspect (Fig. 1E: M-S, Fig. 1F: L-S), with lateral slices containing barrels corresponding to ventral whiskers and medial slices containing barrels corresponding to dorsal whiskers (Petersen et al., 2020). In L-S slices, however, the slice plane becomes nearly tangential, so these slices were excluded.

To evaluate the slice alignment illustrated in Fig. 1, we sampled the Brain Globe 3D atlas (Claudi et al., 2020) in 12 locations relevant to each group of slices to estimate the probability *P(row)* of adjacent barrels being in the same row. We obtained *P(row)* = 0.90 in R-C slices, 0.50 in C-C slices, and 0.58 in M-S slices, while P(*arc)* = 0.06, 0.40, 0.17 (note *P(row)* + *P(arc)* + *P(diagonal)* = *1*). We originally intended to associate coronal slices with arcs and sagittal slices with rows. However, this atlas analysis suggests that our sections align well to foveal and exploratory whisking patterns. R-C and C-C slices map onto exploratory whisking, in which protraction is angled in the rostral-dorsal direction. Meanwhile, the sagittal plane can be used to track foveal whisking, in which protraction tends toward the rostral-ventral direction (Knutsen et al., 2005; Petersen et al., 2020). This correspondence is a consequence of the angular offset between barrel and whisking fields. Our alignment allows us to relate these whisker field axes to inter-barrel communication.

bioRxiv preprint doi: https://doi.org/10.1101/2025.11.04.686617; this version posted January 12, 2026. The copyright holder for this preprint (which was not certified by peer review) is the author/funder, who has granted bioRxiv a license to display the preprint in perpetuity. It is made available under a CC-BY 4.0 International license.

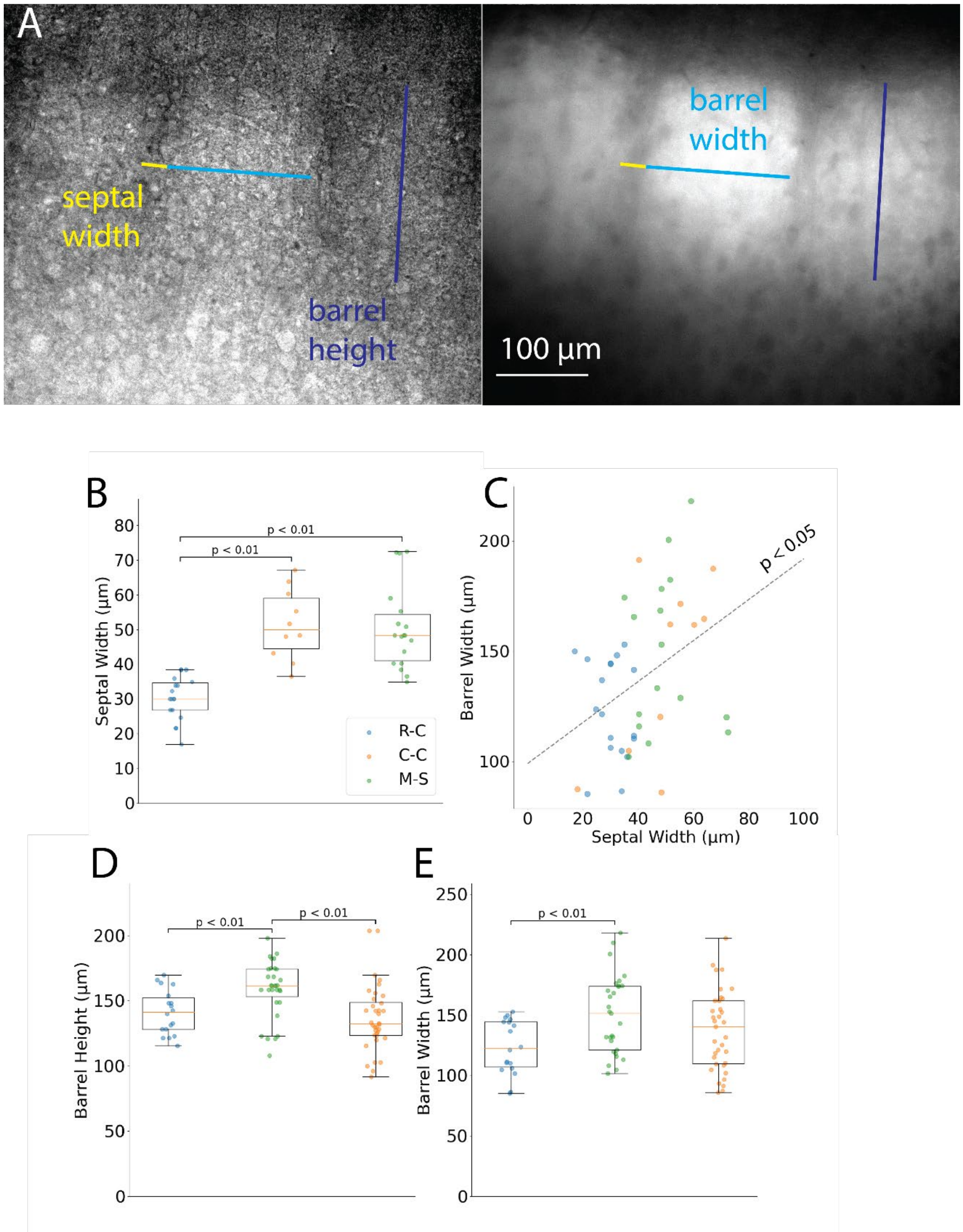

Fig. S1: **Barrel dimensions across slice types. A.** Bright field (left) and resting fluorescence (right) images featuring barrel width (light blue) and height (dark blue), and septum width (yellow). The L2/3-L4 boundary is determined based on the presence of septal columns and higher fluorescence in L4 than in L2/3. **B**. Comparison of septal width among R-C, C-C, and M-S slices (note that L-S slices were not included, see text). **C**. Plot of barrel width versus septum width with line from linear regression ($p < 0.05$). **D-E.** Barrel height and width differ across slice types (M-S barrel height is greater than that of R-C and C-C; R-C barrels were

bioRxiv preprint doi: https://doi.org/10.1101/2025.11.04.686617; this version posted January 12, 2026. The copyright holder for this preprint (which was not certified by peer review) is the author/funder, who has granted bioRxiv a license to display the preprint in perpetuity. It is made available under a CC-BY 4.0 International license.

narrower than M-S barrels). Each point indicates one barrel or septum; lines show mean ± SEM. Significance assessed by Welch's t-test.

Barrel dimensions

The clear boundaries highlighted by Scnn1a labeling enabled us to estimate the basic barrel dimensions. The septal width, barrel width, and barrel height, annotated on bright-field (left) and fluorescence (right) images in Fig. S1A, were estimated from the cytoarchitectural boundaries. We distinguished L2/3 from L4 based on a steep fluorescence gradient and the loss of distinction between septa and barrel hollows at the boundary. Fig. S1B compares septal widths between the R-C, C-C, and M-S slices. The mean septal width in the rostral row of R-C slices, 30.3 ± 1.5 μm (n = 18 septa, 15 slices), was significantly smaller than in C-C slices (51.5 ± 3.2 μm, n = 10 septa, 10 slices; Welch's t-test: T = 5.99, $p = 5x10^{-5}$) or in M-S slices (50.4 ± 2.8 μm, n = 18 septa, 13 slices, T = 6.38, $p = 10^{-6}$). R-C and M-S septal widths did not differ significantly (T = 0.24, p = 0.8). These variations were exploited to track the transition from rostral-aspect (R-C) to caudal-aspect (C-C) in the sequence in which coronal slices were cut. Barrel width and height did not correlate significantly (linear regression, R = 0.19, p = 0.09), which is expected given the heterogeneity of barrel dimensions and the fact that the thickness of L4 limits barrel height to 208 ± 5 μm or less (DeFelipe et al., 2002). However, barrel and septal width showed a significant positive correlation (Fig. S1C, R = 0.38, p = 0.01). Additionally, sagittal slices were cut at a smaller angle (less than a right angle) to the cortex layers, particularly towards the medial aspect but also in the lateral aspect. As a result, L4 is thicker in some sagittal slices and barrel height is significantly greater in M-S slices (Fig. S1D, 159 ± 4 μm, n = 31 barrels) than in R-C slices (141 ± 4 μm, n = 18, T = 3.1, p = 0.003) or in C-C (137 ± 4 μm, n = 36, T = 3.9, p = 0.0003). Barrel widths were significantly smaller in R-C slices (Fig. S1E, 124 ± 5 μm, n = 18) than in M-S slices (150 ± 6 μm, n = 29, T = 3.33, p = 0.002), but not significantly different from C-C slices (138 ± 6 μm, n = 35, T = 1.55, p = 0.13). These differences in barrel width match the trend in barrel size seen in the tangential view of BC, in which barrels become smaller in the rostro-medial aspect and have smaller cross-sections in the coronal plane (Fig. 1B, lower right; (Petersen et al., 2020). Our choice of 200 μm slice thickness limits background from barrels outside of the microscope focal plane, given the sum of our measurements of barrel width plus septal width (154-200 μm).

Spatiotemporal biases in inter-barrel communication: isotropic response properties

In Fig. S2A, comparisons of mean latency of neighbor barrel responses showed no significant differences between planes (Fig. 8E, Welch's t-test, T = 1.7, p = 0.11) or between protraction and retraction phases (T = 0.63, p(32) = 0.27).

In Fig. S2B, the contrast model showed no significant difference in half-width between protraction and retraction directions (T = 1.0, p(32) = 0.16), nor between coronal and sagittal slices (Welch's t-test, T = 0.75, p = 0.46).

Direction did not significantly affect neighbor/home ΔF/F ratio, nor did slice plane (Welch's t-test, coronal: 0.36 ± 0.05; sagittal: 0.31 ± 0.05; T = 0.67, p = 0.51) or phase (Fig. S2C; sum contrast model, T = 1.0, p(32) = 0.15).

bioRxiv preprint doi: https://doi.org/10.1101/2025.11.04.686617; this version posted January 12, 2026. The copyright holder for this preprint (which was not certified by peer review) is the author/funder, who has granted bioRxiv a license to display the preprint in perpetuity. It is made available under a CC-BY 4.0 International license.

Grouped by slice plane, the smaller mean coronal velocity (117 ± 5 µm/ms, n = 29) was not significantly different from the mean sagittal velocity of 133 ± 14 µm/ms, n = 12 (Fig. S2D; T = 1.1, p = 0.30). With its more specific test, the contrast model showed that protraction velocities were not significantly smaller than retraction velocities (T = 0.12, p(36) = 0.45).

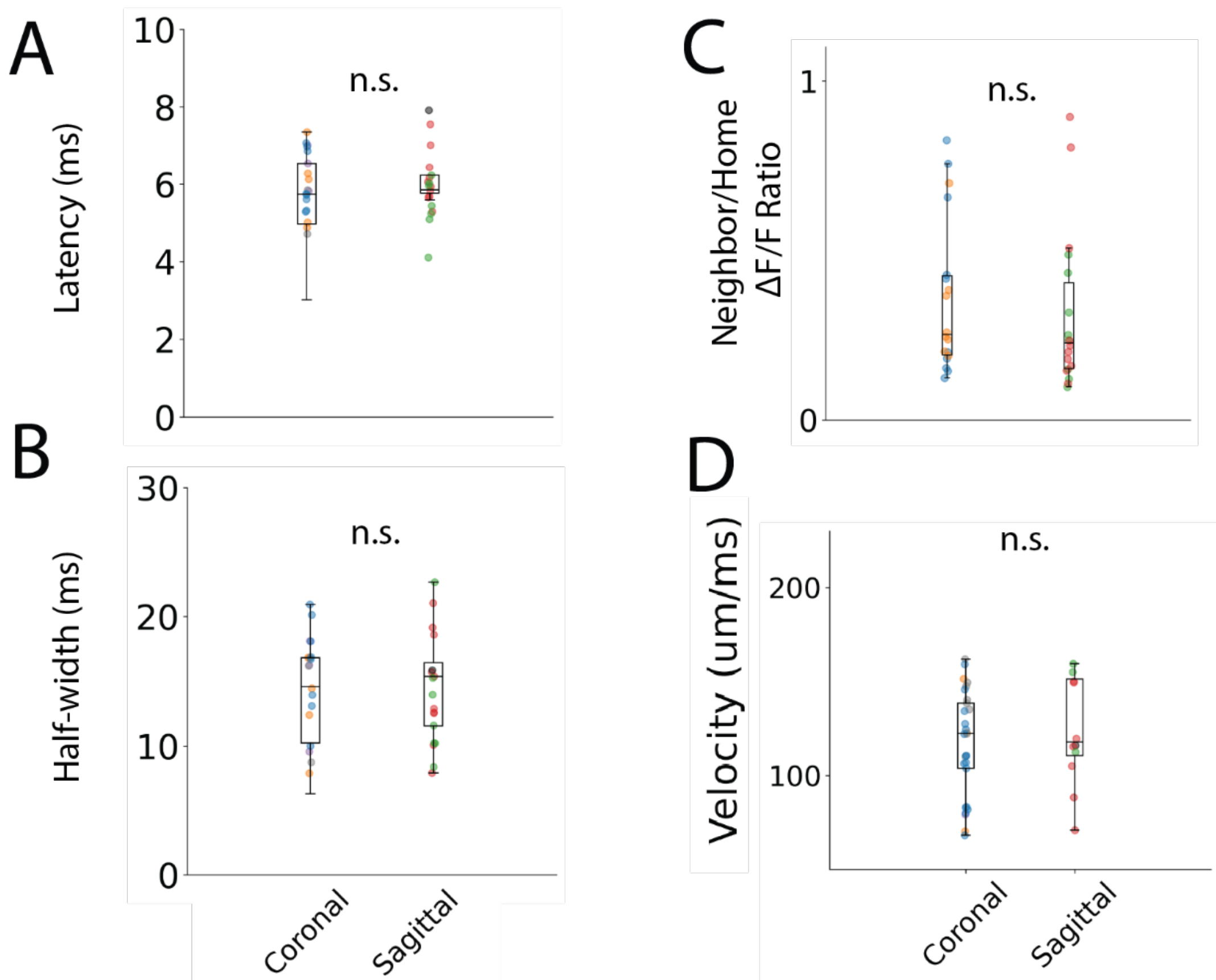

Fig. S2: **Inter-barrel response properties are symmetric in coronal and sagittal slices.** Latency (**A**), half-width (**B**), neighbor/home ΔF/F ratio (**C**) and velocity (**D**) of neighbor barrel responses grouped by slice plane: coronal or sagittal. Colors denote Caudal in M-S slices, Rostral in M-S slices, Dorsal in C-C slices, Ventral in C-C slices, Dorsal in R-C slices, and Ventral in R-C slices; R-C and C-C of the same direction are grouped in boxplots and scatter plots. Boxplots show minimum, first quartile, median, third quartile, and maximum with scatter; means ± SEM reported in Results. Statistical analysis used Welch's t-tests as appropriate.

Velocity is unaffected by AMPA-receptor blockade or synaptic depression

The mean velocity within L4 neighbor barrels was similar regardless of stimulation site (Fig. S3A, open circle markers; L2/3: 129 ± 12 µm/ms, L4: 122 ± 5 µm/ms, T = 0.98, p = 0.36). L4 neighbor velocities were computed for the minority of cases where NBQX did not completely abolish responses (ACSF: n = 31 responding L4 neighbor barrels, NBQX: n = 6). NBQX had no significant effect on velocity (Fig. S3A, bordered markers; ACSF and NBQX, L2/3: T = 1.16, p = 0.27, L4: T = 1.0, p = 0.34). This suggests that axonal conduction speed (antidromic or orthodromic) determines the velocities of response spread, despite the biphasic nature of many responses in neighboring barrels (Fig. 7D, top traces). Velocities were greater in the home barrel (132 ± 10 µm/ms) than the neighboring barrel with both L2/3 or L4 stimulation (mean: 123 ± 6 µm/ms), but this difference was not significant (T = 0.77, p = 0.45, Fig. S3B).

bioRxiv preprint doi: https://doi.org/10.1101/2025.11.04.686617; this version posted January 12, 2026. The copyright holder for this preprint (which was not certified by peer review) is the author/funder, who has granted bioRxiv a license to display the preprint in perpetuity. It is made available under a CC-BY 4.0 International license.

Depression in amplitude was not accompanied by a change in half-width (Fig. S3C, home: T = 0.14, p = 0.89; neighbor: T = 0.66, p = 0.51). Additionally, there was no change in conduction velocity from the first to second pulse (Fig. S3D, 99 ± 9 µm/ms to 98 ± 8 µm/ms, T = 0.11, p = 0.91), suggesting a constant speed for processing information between adjacent barrels.

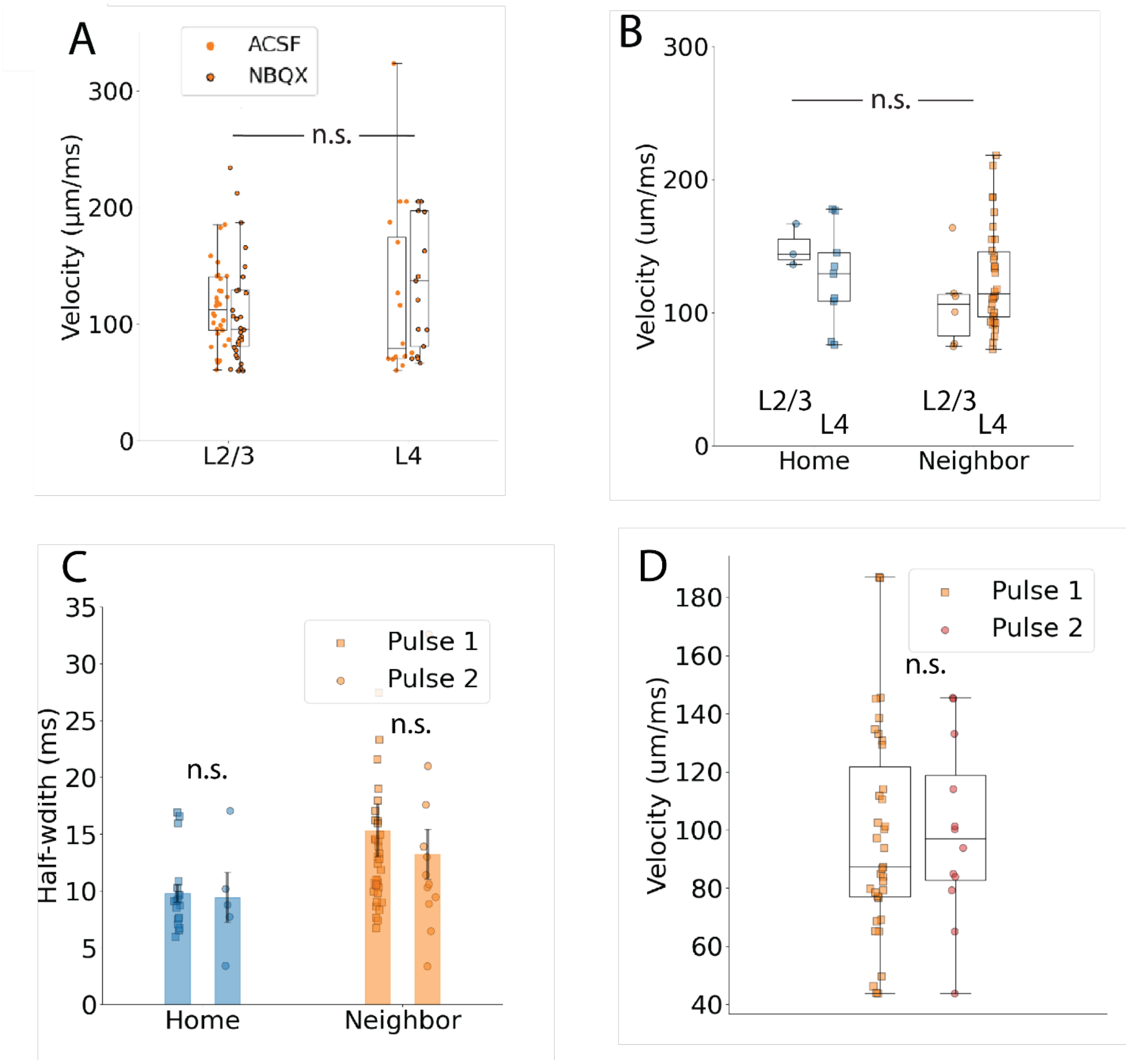

Fig. S3: **Velocity and half-width persist across manipulations A**. Boxplot and scatter of velocity measurements before and after NBQX. NBQX had no significant effect. **B.** Velocity was greater in the home barrel but not significantly different from neighbor barrel velocities. **C.** No significant change in velocity between the first and second responses. **D.** Response half-width was also unchanged. Note that the difference between neighbor and home half-widths was discussed in regard to Fig. 3H, so we have left it unmarked here to avoid repetition of results and to focus on the present comparison of first and second responses. Boxplots show minimum, first quartile, median, third quartile, and maximum with scatter; means ± SEM reported in Results. Statistics used barrel means as independent samples, although individual ROI measurements are displayed.

bioRxiv preprint doi: https://doi.org/10.1101/2025.11.04.686617; this version posted January 12, 2026. The copyright holder for this preprint (which was not certified by peer review) is the author/funder, who has granted bioRxiv a license to display the preprint in perpetuity. It is made available under a CC-BY 4.0 International license.

Age and sex do not affect response properties of the present study

A comparison of male versus female mice showed no significant differences in latency, half-width, velocity (Welch's t-test, $T < 0.001$, $p = 1.0$ for all), or paired-pulse ratio (PPR) (multivariate linear regression, $F=0.55$, $p = 0.46$). Mice aged 30 to 60 days showed no significant correlation between these metrics and age: response latency, half-width (linear regression, L2/3: latency: $p = 0.23$, half-width: $p = 0.48$; L4: latency: $p = 0.16$, half-width: $p = 0.57$), velocity (linear regression, $p = 0.66$) or PPR (multivariate linear regression, $F = 0.006$, $p = 1.0$).

bioRxiv preprint doi: https://doi.org/10.1101/2025.11.04.686617; this version posted January 12, 2026. The copyright holder for this preprint (which was not certified by peer review) is the author/funder, who has granted bioRxiv a license to display the preprint in perpetuity. It is made available under a CC-BY 4.0 International license.

Supplementary Material References

Claudi, F., Petrucco, L., Tyson, A., Branco, T., Margrie, T., & Portugues, R. (2020). BrainGlobe Atlas API: a common interface for neuroanatomical atlases. *Journal of Open Source Software*, *5*(54), 2668. https://doi.org/10.21105/joss.02668

DeFelipe, J., Alonso-Nanclares, L., & Arellano, J. I. (2002). Microstructure of the neocortex: Comparative aspects. *Journal of Neurocytology*, *31*(3-5), 299-316. https://doi.org/10.1023/a:1024130211265

Knutsen, P. M., Derdikman, D., & Ahissar, E. (2005). Tracking Whisker and Head Movements in Unrestrained Behaving Rodents. *Journal of Neurophysiology*, *93*(4), 2294-2301. https://doi.org/10.1152/jn.00718.2004

Lazic, S. E. (2010). The problem of pseudoreplication in neuroscientific studies: is it affecting your analysis? *BMC Neuroscience*, *11*(1). https://doi.org/10.1186/1471-2202-11-5

Petersen, R. S., Colins Rodriguez, A., Evans, M. H., Campagner, D., & Loft, M. S. E. (2020). A system for tracking whisker kinematics and whisker shape in three dimensions. *PLOS Computational Biology*, *16*(1), e1007402. https://doi.org/10.1371/journal.pcbi.1007402
